# Sinter‐ and Water‐Resistant Pt Enabled by High Entropy of Porous Oxide Nanofibers

**DOI:** 10.1002/advs.202501334

**Published:** 2025-04-25

**Authors:** Yunpeng Wang, Mingyu Tang, Zhuxin Lyu, Wanlin Fu, Han Yan, Shiming Zhou, Yueming Sun, Yunqian Dai

**Affiliations:** ^1^ School of Chemistry and Chemical Engineering Southeast University Nanjing Jiangsu 211189 P. R. China; ^2^ Hefei National Research Center for Physical Sciences at the Microscale University of Science and Technology of China Hefei Anhui 230026 P. R. China

**Keywords:** CO oxidation, high‐entropy oxide, nanofibers, Pt, sinter‐resistance

## Abstract

Supported ultrafine noble metal species, especially for Pt, suffer from inevitable sintering at temperatures as low as 80 °C, severely limiting their stability and thus their practical applications. In this work, a strategy is demonstrated using the high‐entropy effect to prevent sub‐2.6 nm Pt nanoparticles from sintering. Due to the higher mixing entropy and thus lower Gibbs free energy of porous high‐entropy oxide (HEO) nanofibers in the catalytic system, the supported Pt remained thermally stable up to 1000 °C, as verified by in situ HAADF−STEM observation. Even after being hydrothermally aged with 10 vol% vapor at 850 °C, this catalytic system maintained the Pt size of 2.9 nm, demonstrating remarkable sinter‐resistance and water tolerance. Particularly, after aging at 850 °C, the Pt/HEO catalytic system maintained its full CO conversion for 338 h without any decline. These results highlight the positive effect of increasing configurational entropy on the thermal stability of the entire catalytic system, providing a reliable solution for catalytic conversions involving high temperatures.

## Graphical abstract

The high‐entropy effect thermodynamically favors a unique distribution of multiple constituent elements within the crystalline lattice with low Gibbs free energy. This special configuration endows the material with exceptional stability, depressing the growth of Pt species at elevated temperatures. Besides, the high‐entropy oxide has a significantly higher H_2_O dissociation energy and suppresses water adsorption, enabling improved water tolerance.

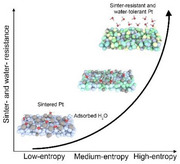

## 1 Introduction

With the global rise of advanced nanocatalysts to address environmental and energy challenges, sinter‐resistance is crucial for their practical applications.^[^ ^1^ ^]^ To enhance catalytic activity and stability, reactive metal species are typically reduced to the nanoscale, thereby increasing their specific surface area and consequently exposing sufficient reactive sites. However, as the size of the metal decreases, the increased surface energy causes the Tammann temperature to decrease (i.e., the temperature sintering starts to occur).^[^ ^2^ ^]^ Taking 3‐nm Pt nanoparticles as an example, the Tammann temperature decreases to below 250 °C, extremely lower than that of bulky Pt (i.e., 749 °C).^[^ ^3^ ^]^ Above the Tammann temperature, metals are prone to sintering, an irreversible process where particle growth occurs, leading to catalyst deactivation.^[^ ^4^ ^]^ When exposed to steam, the generated volatile metal compounds coordinated with water commonly cause accelerated sintering.^[^ ^5^ ^]^ To address this issue, porous oxides have emerged as an ideal support for dispersing catalytic metal species,^[^ ^6^ ^]^ as they can spatially confine metal migration within the porous matrix.^[^ ^7^ ^]^ However, the large surface area of porous oxides also drives them to collapse and densify to minimize surface free energy at high temperatures.^[^ ^8^ ^]^ Therefore, maintaining the porous structure within the solid support is of great scientific and technological importance.

High‐entropy oxides (HEOs) represent a novel class of metal oxides, which are defined as five or more near‐equimolar metal elements arranged deliberately in a single‐phase lattice with randomized distribution.^[^ ^9^ ^]^ Multiple element distribution at the atomic level results in a high‐entropy system with improved mixing entropy, lowering the Gibbs free energy of the whole system and making the material less prone to phase transitions when heated.^[^ ^10^ ^]^ Moreover, the multi‐electron redox properties, as well as the dynamic tunability between entropy and surface energy, make HEO a potential support for stabilizing metal species.^[^ ^11^ ^]^ Currently, the most common approaches to fabricating HEO are ball‐milling and co‐precipitation, which lack precise control over the fine structure within the resultant HEO. In addition, the obtained HEO nanostructures are subjected to coalescence over time during practical applications, resulting in a gradual reduction of active sites and consequent decline in catalytic activity.^[^ ^12^ ^]^ Electrospinning, a versatile and simple technique for generating ultrathin nanofibers, provides a reliable approach for producing HEO nanofibers, which hold great promise in bridging the gap between the various primary delicate‐designed powders to the final advanced materials.^[^ ^13^ ^]^

In this work, porous HEO nanofibers were elegantly produced via electrospinning technology, remarkably promoting the sinter‐resistance and water‐tolerance of supported sub‐2.6 nm Pt nanoparticles. Due to the high‐entropy effect, the surface free energy of the whole catalytic system was substantially reduced, thereby diminishing the thermodynamic driving force for sintering. As a result, 2.6‐nm Pt remained stable in size at 4.2 nm after aging even at 950 °C and maintained a size of 2.9 nm after aging at 850 °C in a humid atmosphere. In situ, HAADF−STEM revealed that the HEO nanofibers kept their nanopore structures up to 1000 °C. Therefore, the sinter‐resistant and water‐tolerant Pt/HEO catalytic system maintained 100% CO conversion for more than 338 h and withstood 35‐cycles reaction without declining even in the presence of vapor. This work reveals new insights into the high entropy‐driven sinter‐resistance of Pt species, offering new perspectives for exploring advanced catalytic systems with boosted durability.

## 2 Results and Discussion

### 2.1 Design and Fabrication of Ultra‐Stable Porous HEO Nanofibers

**Scheme 1:**
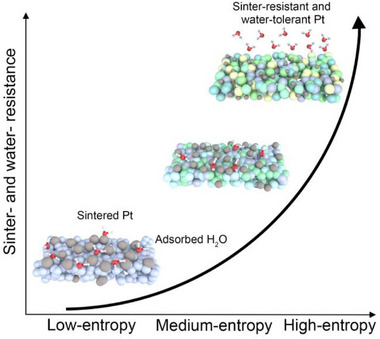
High‐entropy driven stability of Pt species on porous oxide nanofiber with sinter‐resistance and water‐tolerance.

To stabilize Pt species against sintering, high‐entropy porous nanofibers with reliable thermal stability were developed. A positive correlation between increasing configurational entropy and catalytic stability was demonstrated, as illustrated in **Scheme** **1**. The high‐entropy effect thermodynamically favors a unique distribution of multiple constituent elements within the crystalline lattice with low Gibbs free energy. This special configuration endows the material with exceptional stability, depressing the growth of Pt species at elevated temperatures. Besides, the high‐entropy oxide has a significantly higher H_2_O dissociation energy and suppresses water adsorption compared with single metal oxides,^[^ ^9a^ ^]^ enabling improved water tolerance. Therefore, metal species within such catalytic systems can be endowed with boosted sinter‐resistance and water tolerance at high temperatures.

**Figure 1:**
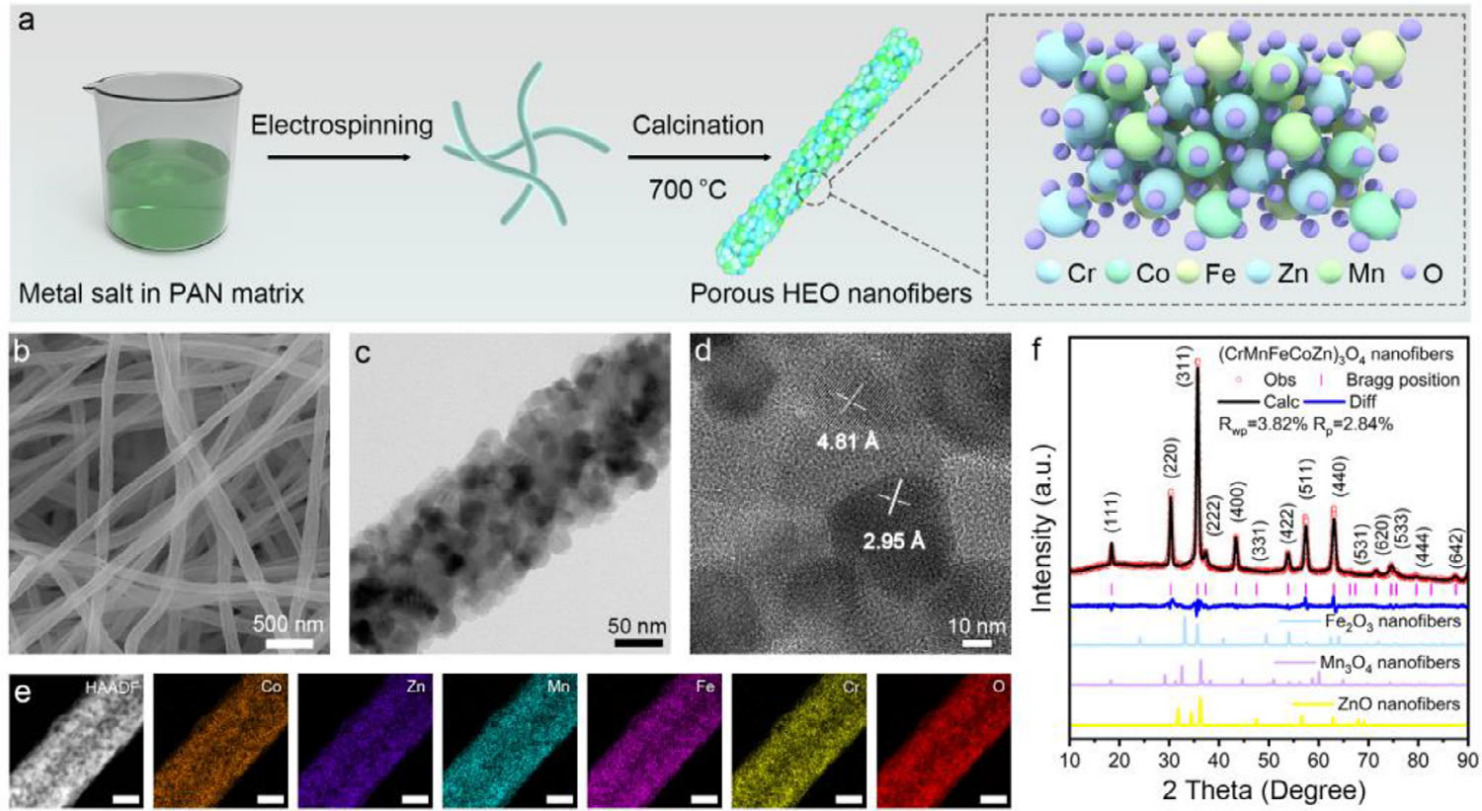
Structural characterizations of (CrMnFeCoZn)_3_O_4_ high‐entropy oxide (HEO) nanofibers. a) Schematic illustration for electrospinning HEO nanofibers. b) SEM, c) TEM, d) high‐resolution TEM, and e) HAADF−STEM and elemental mapping images of HEO nanofibers. The scale bars in (e) are 50 nm. f) XRD patterns of Fe_2_O_3_, Mn_3_O_4_, ZnO, and (CrMnFeCoZn)_3_O_4_ HEO nanofibers. Rietveld refinement shows data points (red circle), the calculated intensity (black line), the positions of reflection peaks (pink vertical bar), and the difference profile (blue line).

The porous HEO nanofibers were fabricated by electrospinning a spinnable precursor containing five metal salts (Cr, Mn, Fe, Co, and Zn) and polyacrylonitrile (PAN), followed by calcination at 700 °C in air (**Figure** **1**a). As the typical SEM image (Figure 1b; Figure S1, Supporting Information) shows, the obtained HEO nanofibers are uniform in diameter with an average diameter of only 140 nm (Figure S2, Supporting Information). Moreover, TEM observation reveals that the nanofibers exhibit a porous structure composed of densely packed 10‐nm oxide grains (Figure 1c). As the high‐resolution TEM image depicted in Figure 1d, lattice distances of 4.81 and 2.95 Å were observed on HEO nanograins, corresponding to the (111) and (220) planes of spinel‐type oxides respectively.^[^ ^14^ ^]^ The high‐angle annular dark‐field scanning transmission electron microscopy (HAADF−STEM) and elemental mapping provided evidence that the Cr, Mn, Fe, Co, Zn, and O elements are uniformly distributed throughout an individual nanofiber, proving the generation of HEO nanofibers (Figure 1e; Figure S3, Supporting Information).

Rietveld refinement of XRD data (Figure 1f) confirms the phase purity and crystallinity of (CrMnFeCoZn)_3_O_4_ nanofibers, with diffraction peaks at 18.4°, 30.3°, 35.7°, 43.4°, 53.8° and 63.0° corresponding to the (111), (220), (311), (400), (422) and (440) planes of the spinel structure, respectively (CoCr_2_O_4_, JCPDS 78−0711).^[^ ^15^ ^]^ The refinement results (*R*_wp_ = 3.82%, *R_p_* = 2.84%) indicated a spinel structure with good phase purity and a random distribution of five metal elements (i.e., Cr, Mn, Fe, Zn, Co) in the lattice, generating high configurational entropy.^[^ ^16^ ^]^ The crystallite size, estimated via the Scherrer formula, is ≈11.4 nm, consistent with TEM observations of interconnected grains.

The carefully selected PAN matrix, with high decomposition temperature, effectively prevents HEO nanograin from overgrowth during calcination at high temperatures. After the removal of PAN via combustion, the metal oxide nanograins were confined in 1D fibrous structures and thus readily transited to HEO nanograins with well‐defined spinel crystalline structure. Meanwhile, the released gases originating from the decomposition of PAN accelerated the generation of abundant nanopores at the grain boundaries.^[^ ^17^ ^]^ As a result, the HEO nanofibers exhibited a high surface area of 65.8 m^2^ g^−1^ (Figure S4, Supporting Information). When aged at 800 °C, the nanopores within HEO nanofibers remain exceptionally stable, demonstrating the thermal stability of the pore structures that resist densification (Figure S5, Supporting Information).

### 2.2 High‐Entropy Driven Thermal Stability of Ultrafine Pt and Nanopores in HEO Nanofibers

**Figure 2:**
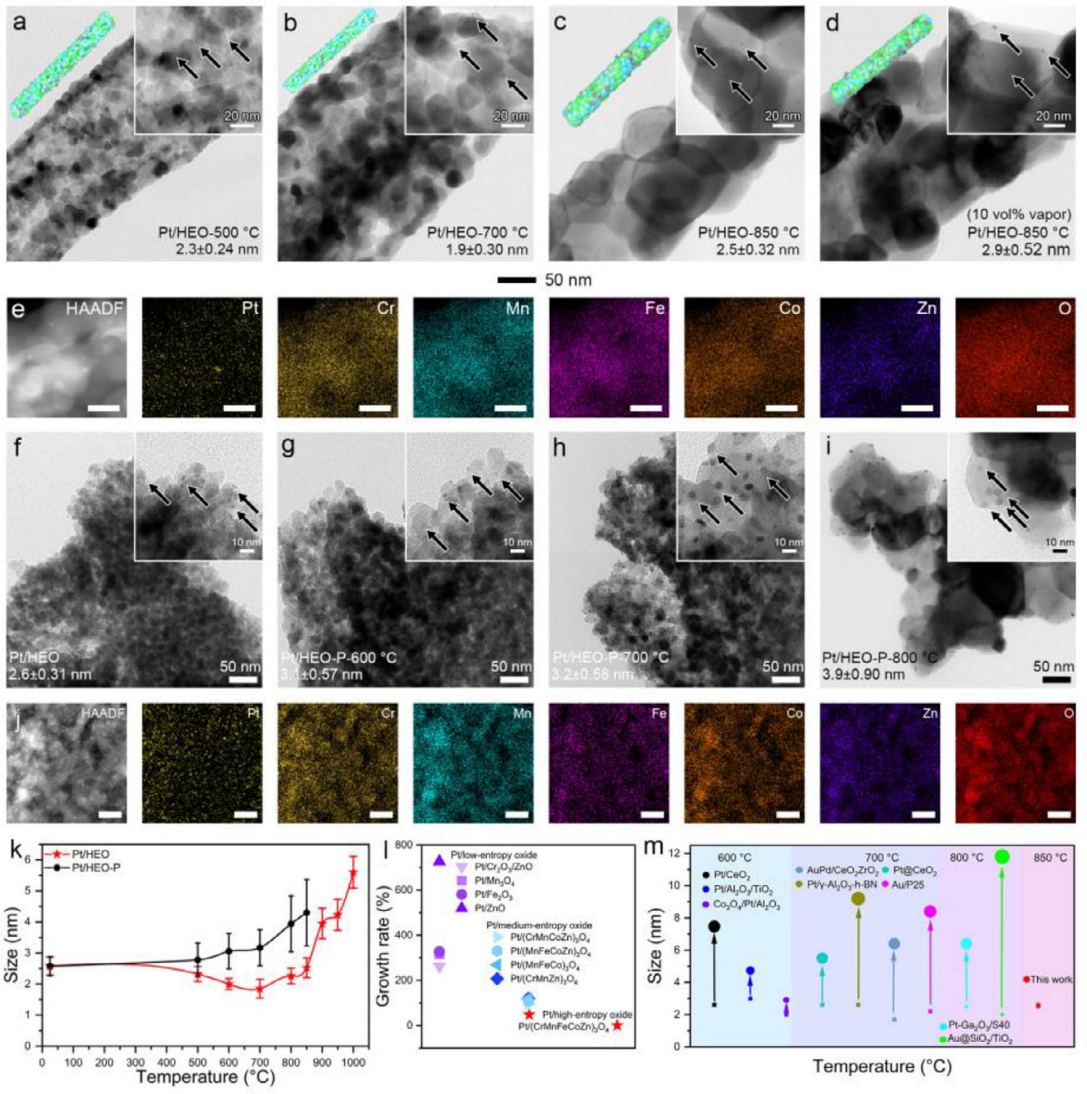
Sinter‐ and water‐ resistant of Pt/HEO. TEM images of a) Pt/HEO‐500 °C, b) Pt/HEO‐700 °C, c) Pt/HEO‐850 °C, d) Pt/HEO‐850 °C (10 vol% vapor). e) HAADF−STEM image and corresponding elemental mappings of Pt/HEO‐700 °C. The scale bars are 20 nm. TEM images of f−i) Pt/HEO‐P at elevated temperatures. j) HAADF−STEM image and corresponding elemental mappings of Pt/HEO‐P‐700 °C. The scale bars are 50 nm. k) Statistics analysis of average Pt nanoparticle sizes at elevated aging temperatures. l) Growth in Pt nanoparticles size after heating at 850 °C of Pt/low‐entropy oxides, Pt/medium‐entropy oxides, and Pt/HEO. m) Comparison of catalyst particle sizes across different metals aged at elevated temperatures.

To explore the potential of the porous HEO nanofibers for stabilizing metal species, 2.6‐nm Pt nanoparticles (0.58 mg mL^−1^), fabricated by a polyol method, were deposited on the HEO nanofibers by an impregnation method (Figure S6, Supporting Information).^[^ ^18^ ^]^ The TEM image reveals a uniform distribution of ultrafine Pt nanoparticles across the entire nanofiber surface. After aging at 500, 600, and 700 °C in N_2_ for 2 h, the size of Pt decreases from 2.6 nm to 2.3, 2.0, and 1.9 nm. Such a decrease in size indicates that the nanoparticles are re‐dispersive on nanofiber surfaces (**Figure** **2**a,b; Figure S7, Supporting Information). A similar size reduction of Pt nanoparticles was observed when aging in air (Figures S8 and S9, Supporting Information). However, at elevated temperatures, the Pt nanoparticles started to grow, reaching 2.3 nm at 800 °C and 2.5 nm at 850 °C (Figure 2c), while becoming smaller than their initial size before aging. The Pt dispersions were determined to be 25% for Pt/HEO‐700 °C, and 20% for Pt/HEO‐850 °C (Figure S10, Supporting Information) via CO chemisorption measurements. These results are consistent with the TEM images, where the Pt nanoparticles in Pt/HEO‐700 °C and Pt/HEO‐850 °C exhibit average diameters of 1.9 and 2.5 nm, respectively. It should be noted that the porous structure of the HEO nanofibers maintained stability up to 850 °C.

In addition to thermal stress, the adsorbed chemicals on both the metal surface and the support can significantly accelerate sintering, potentially through chemical reactions.^[^ ^6b^ ^]^ Specifically, water molecules adsorbed on Pt can coordinate with Pt species, increasing their mobility and thereby posing a significant challenge for stabilizing Pt under humid and high‐temperature conditions. In 10 vol% vapor, Pt/HEO maintained structural stability with minimal sintering: Pt nanoparticles retained sizes of 2.7 nm at 750 °C, 2.8 nm at 800 °C, and 2.9 nm even at 850 °C (Figure 2d; Figure S11, Supporting Information). This observation suggests the addition of vapor shows an ignorable impact on the thermal stability of Pt in the catalytic system, showing reliable water‐tolerance. The HAADF−STEM images confirmed the high dispersion of Pt nanoparticles on the HEO surface, with uniform distribution of Cr, Mn, Fe, Co, Zn, and O (Figure 2e). HRTEM revealed clear lattice fringes with interplanar distances of 4.71 Å, corresponding to the (200) plane of the HEO (Figure S12, Supporting Information).

Moreover, the Pt loading can be easily increased from 0.4 to 1.3 wt.% by simply changing the PAN to polyvinylpyrrolidone (PVP) matrix during electrospinning, as the resultant HEO (denoted as HEO‐P) endows high porosity and favorable zeta‐potential for Pt loading (Figure S13, Table S1, Supporting Information). Notably, the HEO‐P sample maintains an enhanced surface area of 75.3 m^2^ g^−1^ (Figure S14, Supporting Information). With such a high Pt loading, the Pt/HEO‐P also maintained remarkable thermal stability. After aging, the closely dispersed Pt slightly grows to 2.8, 3.1, 3.2, 3.9, and 4.3 nm at 500, 600, 700, 800, and 850 °C (Figure 2f–i; Figures S15–S17, Supporting Information). HAADF−STEM analysis revealed highly dispersed Pt nanoparticles on the HEO surface and a uniform distribution of Cr, Mn, Fe, Co, Zn, and O, demonstrating exceptional sinter‐resistance (Figure 2j). Even at high heating temperatures, the size of the Pt nanoparticles remains small (Figure 2k).

These phenomena reveal that the nanoparticles and pore structure of the support are long‐term stable at 850 °C. Above 900 °C, the support transitioned from a porous structure to a dense chain‐like morphology, leading to a reduction in pore density and surface area, which allowed for the growth of Pt nanoparticles. However, at temperatures of 900 °C, 950 °C, and 1000 °C, the sizes remained at 4.1, 4.2, and 5.6 nm, respectively (Figures S18 and S19, Supporting Information). In practical applications, high‐temperature catalytic reactions, such as steam methane reforming and petroleum cracking, require catalysts that remain stable at 1000 °C in some cases. This advancement enhances the suitability of catalysts for applications in extreme environments. Notably, XRD analysis revealed no change in the crystal structure of HEO spinel nanofibers at elevated temperatures despite of a gradual increase in crystallite sizes (Figure S20, Supporting Information). The comprehensive Rietveld refinements of the XRD patterns for Pt/HEO and Pt/HEO‐P after high‐temperature aging have been conducted, as detailed in Figure S21 (Supporting Information). The refinement results demonstrate crystallographic stability with *R_p_* and *R_wp_* values below 5%.

XPS measurements were used to examine the chemical states of each element of Pt/HEO aged at various temperatures under N_2_. The lattice oxygen (O*_a_*, blue regions), oxygen vacancy (O*_b_*, green regions), and chemically adsorbed oxygen species on the surface (O*_c_*, such as O2− 2, O^−^, OH^−^, brown regions) were all demonstrated to exist by the O *1s* spectra (Figure S22, Supporting Information).^[^ ^19^ ^]^ Semiquantitative analysis showed that the percentage of oxygen vacancies decreased from 34.2% at 700 °C to 21.0% and 20.7% at 850 °C. Initially, the Fe valence state exhibited a mixture of Fe^2+^ and Fe^3+^. After aging, the valence of Fe showed a decrease. The Fe *2p* XPS spectrum revealed four main peaks: 712.4 and 725.7 eV for Fe^3+^ *2p*_3/2_ and Fe^3+^ *2p*_1/2_, respectively. 710.5 and 723.3 eV for Fe^2+^ *2p*_3/2_ and Fe^2+^ *2p*_1/2_, respectively (Figure S23, Supporting Information).^[^ ^20^ ^]^ For the Cr element, the valence state remains unchanged. Peaks at 576.0 eV correspond to Cr^3+^ *2p*_3/2_ (Figure S24, Supporting Information).^[^ ^21^ ^]^ The valences of Mn and Co showed an initial decrease followed by an increase (Figures S25 and S26, Supporting Information). The Mn *2p* XPS spectrum exhibited two spin‐orbit doublet peaks at 642.2 and 654.4 eV, assigned to Mn *2p*_3/2_ and Mn *2p*_1/2_ signals of Mn^3+^, respectively.^[^ ^22^ ^]^ The peak at 780.3 eV was attributed to Co^3+^, while the characteristic peak at 782.2 eV was attributed to Co^2+^. The Zn element consistently maintained a Zn^2+^ state, with peaks at 1021.1 and 1044.3 eV (Figure S27, Supporting Information).^[^ ^23^ ^]^

The Pt *4f* core level reveals three major components: Pt^0^, Pt^2+^, and Pt^4+^. In the Pt/HEO, the distribution is 45.6% Pt^0^, 28.9% Pt^2+^, and 25.5% Pt^4+^, corresponding to metallic Pt nanoparticles and ionic interfacial species interacting with the HEO (Figure S28, Supporting Information).^[^ ^24^ ^]^ Following N_2_ treatment, the Pt^0^ significantly declines to 12.3%, while the Pt^2+^ increases to 34.4% in the Pt/HEO‐700 °C, indicating that Pt bonds with surface lattice oxygen to form Pt−O−M (where M is Cr, Mn, Fe, Co, or Zn) surface species.^[^ ^25^ ^]^ Additionally, the redispersion of Pt nanoparticles is confirmed by a rise in Pt^4+^ to 53.3%.^[^ ^26^ ^]^ After further aging at 850 °C, the Pt^0^ increases to 38.7% due to the formation of larger sintered nanoparticles. This trend suggests that the high‐entropy effect can boost the chemical interactions between Pt and HEO, therefore improving the thermal stability of the whole Pt/HEO catalytic system.

Low‐entropy and medium‐entropy oxide nanofibers were prepared by calcining PAN‐based composite nanofibers after selectively removing one or more element(s) (Figures S29 and S30, Supporting Information). We find that the grain size of nanofibers increases with the decrease of configurational entropy. This phenomenon demonstrates that the thermal stability of HEO is significantly enhanced by increasing configurational entropy in the catalytic system. Additionally, the morphological integrity and structural uniformity are improved by transitioning from low‐entropy to medium‐entropy oxide materials. Specifically, by rationally designing the constituent elements, the synergistic effects between various elements are fully realized, effectively inhibiting the growth and agglomeration of grains under high‐temperature conditions. Therefore, the thermal stability of Pt nanoparticles and porous structure significantly declines without the high‐entropy effect. The Pt nanoparticles aggregated on Fe_2_O_3_, Mn_3_O_4_, ZnO, Cr_2_O_3_/ZnO, (MnFeCo)_3_O_4_, (CrMnCo)_3_O_4_, (CrMnCoZn)_3_O_4,_ and (MnFeCoZn)_3_O_4_ nanofibers, highlighting the critical role of the high‐entropy effect (Figures S31 and S32, Supporting Information). After heating at 850 °C, the pore structure of the nanofiber almost disappeared. The size of Pt nanoparticles shows a clear decreasing trend with increasing configurational entropy. Specifically, the size reduces from 21.5 nm for Pt/ZnO‐850 °C, to 9.4 nm for Pt/Cr_2_O_3_/ZnO‐850 °C, further to 5.7 nm for Pt/(MnFeCo)_3_O_4_‐850 °C, and reaches as small as 5.1 nm for Pt/(CrMnCoZn)_3_O_4_‐850 °C, demonstrating the stabilizing effect of the high‐entropy in maintaining Pt nanoparticle stability beyond their Tammann temperature. It is evident that the Pt nanoparticle size grows largest on low‐entropy oxides, followed by medium‐entropy oxides, and the smallest in high‐entropy oxides at the same aging temperature (Figure 2l). The sinter‐resistance of Pt nanoparticles remained high at elevated temperatures, compared with recent reports (Figure 2m; Table S2, Supporting Information).^[^ ^24^, ^26^, ^27^ ^]^ The remarkable stability of the high‐entropy system, particularly in a high Pt loading, mainly originates from the high‐entropy effect. This unique configuration endows the material with exceptional structural stability, effectively preventing the aggregation of Pt species during catalytic reactions.

### 2.3 Dynamic Observation of the Thermal Stability of Pt and the Nanopores Within the HEO

**Figure 3:**
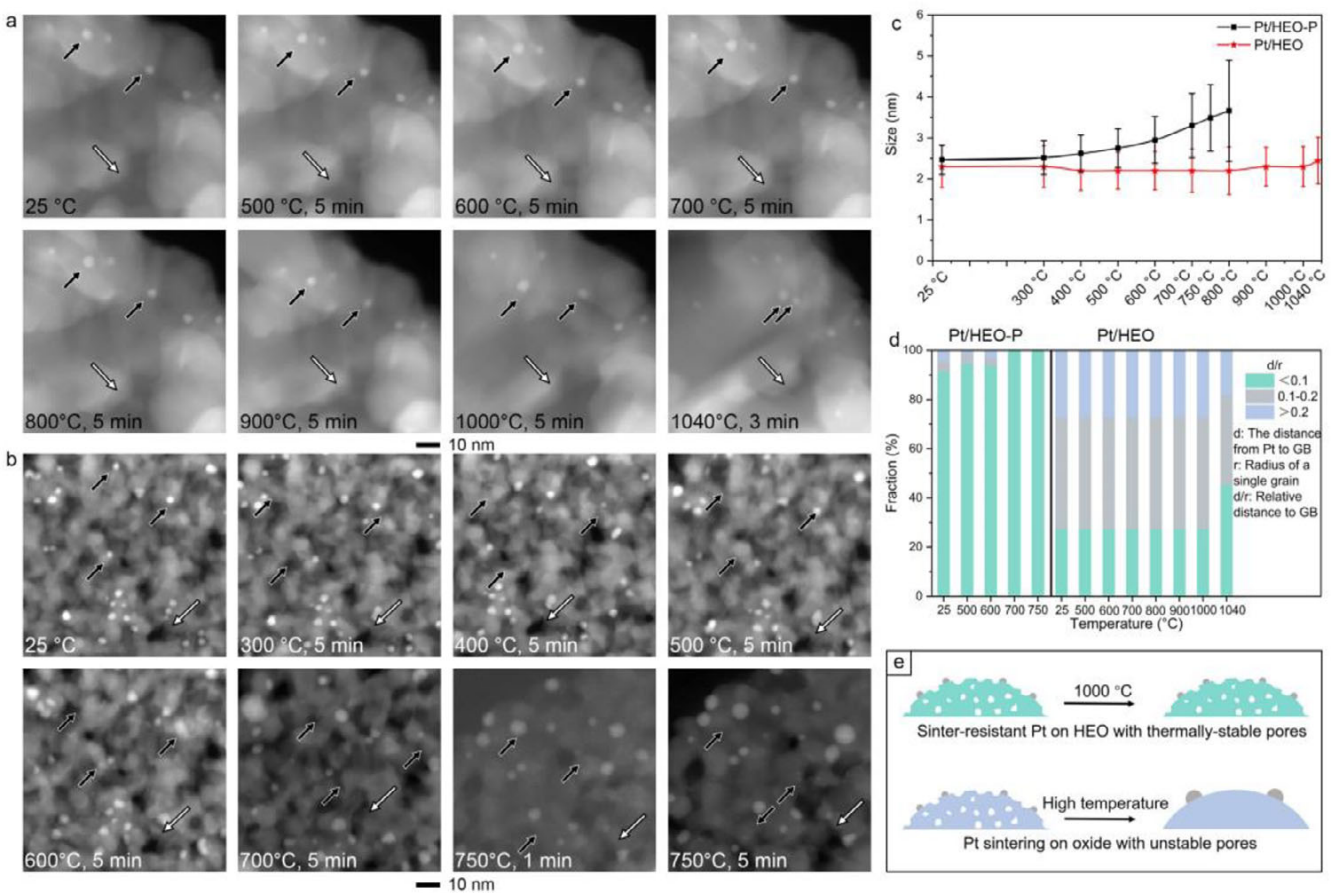
Dynamic observation of the thermal stability of the catalytic system. Time‐sequential HAADF−STEM images of a) Pt/HEO and b) Pt/HEO‐P heated at elevated temperatures. c) Summary of the average size of Pt nanoparticles on HEO and HEO‐P nanofibers at elevated temperatures. d) The fractions of Pt nanoparticles located at different relative distances to the grain boundary (GB) for the total number of observed Pt nanoparticles versus the aging temperature. e) Schematic illustration for the stabilization mechanism of Pt nanoparticles over HEO nanofibers.

In situ TEM was conducted to reveal the underlying anti‐sintering mechanism of the Pt/HEO catalytic system (**Figure** **3**a). Remarkably, the HEO nanofibers demonstrated their porous structure even when subjected to in situ heating upon 1000 °C. Typically, inorganic oxide particles undergo growth in size at elevated temperatures, leading to the collapse of the nanopore in a process known as coalescence. This phenomenon significantly reduces surface area and porosity, which obviously affects the performance in various applications. Similarly, the HEO‐P preserved its porous structure even when heated in situ at 700 °C. HEO, composed of multiple metal elements uniformly distributed within the lattice, forms internal high disorder. This complex structure effectively inhibits grain growth and phase separation, enhancing thermal stability at elevated temperatures. Furthermore, the diverse chemical bonding in HEO strengthens structural stability, allowing the porous structure to remain intact at high temperatures.^[^ ^28^ ^]^ This inherent thermal stability of the support ensures that the Pt nanoparticles remain stable at high temperatures. This robust stability is crucial for sustaining catalytic performance in demanding high‐temperature applications.

The Pt nanoparticles over HEO nanofibers remain stable throughout the entire heating range (Figure 3b). By carefully visualizing the support and the change in size of the Pt nanoparticles, the whole sintering process can be divided into two distinct stages. As demonstrated, the support remains stable up to 1000 °C. The size of the Pt nanoparticles exhibits minimal change even within the temperature range of 1040 °C. This thermal stability is crucial for maintaining the structural integrity and functionality of the material in high‐temperature applications. At 1040 °C (the limit of the involved Si_3_N_4_ chip), the support particles undergo partial grain enlargement, which leads to a reduced distance between the Pt nanoparticles, causing them to sinter when they come within the critical sintering range (Figure 3c).

The size of Pt nanoparticles on HEO‐P only slightly increased in the entire heating process. During high‐temperature aging, the relatively low surface energy at the grain boundary (GB) of the HEO causes Pt nanoparticles to spontaneously migrate toward the GB (Figure 3d).^[^ ^29^ ^]^ This diffusion may lead to encounters between individual Pt nanoparticles, resulting in their aggregation into larger nanoparticles. For HEO‐P, the rise in temperature causes the structural collapse somehow. The diminished GB in turn compromises the stability of the Pt nanoparticles. These findings highlight the significant role of the pore structure within HEO nanofibers in suppressing Pt nanoparticle sintering, offering a promising strategy for designing sinter‐resistant nanocatalysts leveraging the high‐entropy effect (Figure 3e).

### 2.4 Catalytic Activity and Stability Assessment of Pt/HEO

CO is an asphyxiating gas toxic to humans at concentrations exceeding 0.1%.^[^ ^30^ ^]^ The primary sources of CO emissions are motor vehicle exhaust and industrial flue gas, produced by the incomplete combustion of fossil fuels.^[^ ^31^ ^]^ Currently, the catalytic oxidation of CO is thought to be the most effective and energy‐efficient technique for purifying CO. The catalytic oxidation of CO is a key heterogeneous gas‐solid process and a valuable probe for studying catalyst structure, metal‒support interactions, adsorption‐desorption behavior, and reaction mechanisms.^[^ ^32^ ^]^

**Figure 4:**
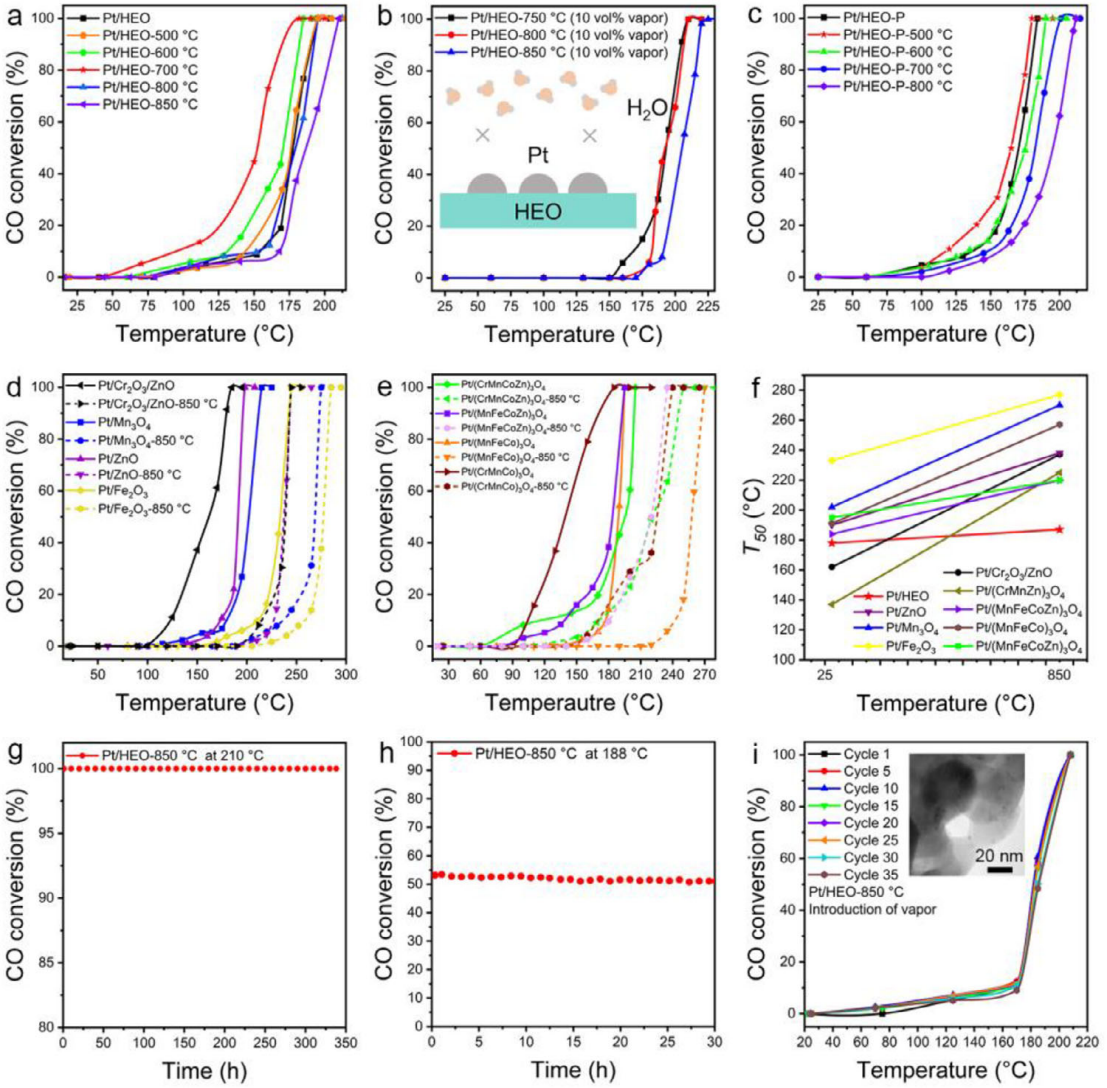
Activity, stability, water‐tolerance of Pt/HEO in CO oxidation. a) CO oxidation performance of Pt/HEO at elevated temperatures. b) CO oxidation performance of Pt/HEO in humid air (10 vol% vapor) at elevated temperatures. c) CO oxidation performance of Pt/HEO‐P at elevated temperatures. d) CO oxidation performance of Pt/Fe_2_O_3_, Pt/Mn_3_O_4_, Pt/ZnO, and Pt/Cr_2_O_3_/ZnO at elevated temperatures. e) CO oxidation performance of Pt/(MnFeCo)_3_O_4_, Pt/(CrMnCo)_3_O_4_, Pt/(MnFeCoZn)_3_O_4_, and Pt/(CrMnCoZn)_3_O_4_ at elevated temperatures. f) *T_50_* of Pt/low‐entropy oxide, Pt/medium‐entropy oxide, and Pt/HEO at elevated temperature. g) The stability of CO oxidation over Pt/HEO‐850 °C. h) The stability of Pt/HEO‐850 °C (10 vol% vapor) was assessed during prolonged operation with sustained 53% CO conversion. i)The cycled CO oxidation measurements over Pt/HEO‐850 °C with the presence of vapor. The inside is a TEM image of Pt/HEO‐850 °C after 36 cycles with the presence of vapor.

The catalytic activity, stability, and water tolerance of the Pt/HEO for CO oxidation were systematically evaluated. As shown in **Figure** **4**a, the Pt/HEO catalyst achieved full CO conversion at 195 °C, significantly lower than the 430 °C required by HEO nanofibers alone (Figure S33, Supporting Information), highlighting the critical role of Pt nanoparticles. Interestingly, the catalytic performance of the Pt/HEO improved substantially after thermal aging. After aging in N_2_ at 700 °C, the conversion temperature dropped to 182 °C. Even after aging at 800 °C, the catalyst maintained high performance. Despite the porous structure structural collapse after aging at 900 °C, the *T_50_* increased by only 37 °C compared to Pt/HEO without aging (Figure S34, Supporting Information). Remarkably, in the presence of 10 vol% vapor, the Pt/HEO maintained structural stability at 850 °C for 2 h, with *T_100_* remaining at 225 °C, which indicates that the catalyst shows rarely good tolerance to vapor (Figure 4b). The low Gibbs free energy of HEO supports the reduced H_2_O adsorption energy and enhanced water tolerance. After aging at 500 °C in air, the catalyst continued to achieve complete CO conversion at a lower temperature of 189 °C, indicating improved activity compared to the pre‐aging condition (Figure S35, Supporting Information). Even when the catalyst was calcined at 600 °C, the temperature required for complete conversion modestly increased to 240 °C. The Pt/HEO‐P demonstrated exceptional stability, maintaining a *T_100_* of 212 °C, only 28 °C higher than its performance without any heating (Figure 4c). This finding highlights the exceptional thermal stability of the Pt nanoparticles supported on HEO nanofibers, making them highly stable and active even under extreme thermal conditions. For these catalysts without high configurational entropy, including Pt/Mn_3_O_4_‐850 °C, Pt/Fe_2_O_3_‐850 °C, Pt/ZnO‐850 °C, Pt/Cr_2_O_3_/ZnO‐850 °C, Pt/(MnFeCo)_3_O_4_‐850 °C, Pt/(CrMnCo)_3_O_4_‐850 °C, Pt/(MnFeCoZn)_3_O_4_‐850 °C, and Pt/(CrMnCoZn)_3_O_4_‐850 °C, *T_100_* values were ≈50 °C higher than those of the catalysts without aging (Figure 4d,e). Figure 4f shows that the increase in *T_50_* of Pt/HEO is much smaller than that of Pt/low‐entropy oxides and Pt/medium‐entropy oxides.

These results underline the exceptional thermal stability of Pt nanoparticles, which is attributed to the high‐entropy effect. As shown in Figure 4g, the Pt/HEO‐850 °C catalyst demonstrated excellent stability during CO oxidation at 210 °C, maintaining consistent activity over 338 h with ignorable decline (Table S3, Supporting Information). The Pt/HEO‐850 °C demonstrates a slight decline (2.4%) in the 30‐h running with a low deactivation rate constant (*k*_d_) of 3.2 × 10^−3^ h^−1^, compared with recent Pt‐based catalysts (Figure 4h).^[^ ^33^ ^]^ This result exceeds the thermal stability conventional catalysts, showcasing its superior sinter‐resistance.

To simulate hydrothermal conditions, vapor was introduced during testing, and even after 35 consecutive cycles, ignorable performance declines were observed (Figure 4i). In the 36th cycle, the porous structure remained intact, with the Pt nanoparticle size increasing only by 0.1–2.7 nm (Figures S36 and S37, Supporting Information). The durability of Pt/HEO in moisture‐rich environments, such as automotive exhaust systems, underscores its potential as a highly reliable catalyst for CO oxidation. The ability of the Pt nanoparticles to resist sintering at elevated temperatures while maintaining catalytic efficiency highlights the robustness of these catalysts, making them highly suitable for applications that demand long‐term thermal stability and resistance to structural degradation.

The Pt/Al_2_O_3_ is a benchmark catalyst for CO oxidation reaction. We prepared Al_2_O_3_ nanofibers via electrospinning, followed by identical Pt nanoparticle loading using the impregnation method. After being hydrothermally aged with 10 vol% vapor at 850 °C, the size of Pt nanoparticles increased significantly from 2.6 to 7.7 nm, accompanied by severe structural collapse of the nanofibers (Figure S38, Supporting Information). CO oxidation performance evaluation revealed a notable degradation in activity and stability: the *T_100_* increased by 70 °C after aging. Furthermore, under prolonged testing at an iso‐conversion condition (59% CO conversion), the catalytic activity declined by 6.4% within 10 h, with a deactivation rate constant of 0.026 h^−1^ (Figure S39, Supporting Information). These results highlight the intrinsic instability of the Pt/Al_2_O_3_ system under hydrothermal conditions, which contrasts with the superior structural and catalytic durability observed in Pt/HEO catalysts.

The atomic‐level dispersion of the support facilitates efficient electron transfer both within the HEO and between the HEO and Pt nanoparticles, protecting the supported Pt from over‐oxidation and reduction.^[^ ^34^ ^]^ This stability prevents significant changes in the valence state of Pt nanoparticles during prolonged reactions, boosting their durability. Once adsorbed on metals, CO combines with lattice oxygen in HEO, which plays a key role in CO oxidation.^[^ ^35^ ^]^ The stability of oxygen species in HEO, combined with efficient electron transfer, ensures that Pt nanoparticles on HEO nanofibers maintain high CO oxidation activity and thermal stability even under harsh conditions. In summary, Pt/HEO provide excellent catalytic performance, thermal stability, and water tolerance, making them highly suitable for demanding applications.

## 3 Conclusion

In this work, we designed an ultra‐stable porous HEO nanofiber for stabilizing Pt nanoparticles with sinter‐resistance and water‐tolerance. Incorporated into the high‐entropy effect of the increased mixing entropy and reduced Gibbs free energy, the 2.6‐nm Pt nanoparticles in the catalytic system exhibited exceptional thermal stability upon various harsh aging. The Pt nanoparticles maintained a size of 4.2 nm even after heating to 950 °C for 2 h under N_2_, above their Tammann temperature. With a high density of Pt nanoparticles (1.3 wt.%), the Pt/HEO maintains a size of 3.9 nm, even after being aged at 800 °C. In situ HAADF‐STEM revealed that the porous HEO support remained stable up to 750 °C, while the Pt nanoparticles started to sinter at 800 °C when the inter‐nanoparticle distance reached the critical sintering range. The Pt/HEO nanofibers demonstrated outstanding thermal stability and water‐resistance toward CO oxidation. Even after being aged at 850 °C, the catalytic system still maintained a stable conversion for over 35 test cycles with vapor and 338 h of reaction. This work provides an alternative way to achieve a sinter‐ and water‐resistant catalytic system, and provides valuable guidance for the development of advanced catalytic systems in the future.

## Conflict of Interest

The authors declare no conflict of interest.

## Supporting information



Supporting Information

## Data Availability

The data that support the findings of this study are available in the supplementary material of this article.
