# Supplementary material for: Sinter‐ and Water‐Resistant Pt Enabled by High Entropy of Porous Oxide Nanofibers
Source: Adv Sci (Weinh). 2025 Apr 25;12(25):2501334. doi: 10.1002/advs.202501334 (PMC12224923; doi:10.1002/advs.202501334)
Supplement: Supplementary file 1 — Supporting Information [file ADVS-12-2501334-s001.docx]

Supporting Information

Sinter- and Water-Resistant Pt Enabled by High Entropy of Porous Oxide Nanofibers

Yunpeng Wang,^a^ Mingyu Tang,^a^ Zhuxin Lyu,^a^ Wanlin Fu,^a^ Han Yan,^b^ Shiming Zhou,^b^ Yueming Sun,^a^ and Yunqian Dai^a,^ *

Experimental Section/Methods

*Materials:* Polyvinylpyrrolidone (PVP, *M*_w_ ≈ 55000 and 1.3 × 10^6^), ethylene glycol, and chloroplatinic acid hydrate (H_2_PtCl_6_·*x*H_2_O, 99.995%) were obtained from Alfa Aesar. Polyacrylonitrile (PAN, *M*_w_≈1.5×10^5^), dimethyl formamide (DMF), (CH_3_COO)_3_Cr (99.9%), (CH_3_COO)_2_Mn·4H_2_O (99.99%), Fe(NO_3_)_3_·9H_2_O (99.9%), (CH_3_COO)_2_Co·4H_2_O (99.9%), (CH_3_COO)_2_Ni·4H_2_O (99.9%), and (CH_3_COO)_2_Zn·2H_2_O (99.9%) were purchased from Macklin. All the chemicals were used as received. The water used in all experiments was filtered through a Millipore filtration system with a resistivity of 18.2 MΩ·cm.

*Fabrication of the porous high-entropy oxide nanofibers:* Porous high-entropy oxide nanofibers were prepared *via* electrospinning. The precursor consisted of 0.7 g of PAN, 5.5 mL of DMF, and 0.5 mmol each of (CH_3_COO)_3_Cr, (CH_3_COO)_2_Mn·4H₂O, Fe(NO_3_)_3_·9H₂O, (CH_3_COO)_2_Co·4H₂O, and (CH_3_COO)_2_Zn·2H₂O. The electrospinning was conducted at a flow rate of 0.3 mL/h and a voltage of 12.5 kV. After calcination in air at 700 °C for 30 min, the composite nanofibers transformed into high-entropy (CrMnFeCoZn)_3_O_4_ oxide nanofibers (HEO nanofibers). To create HEO-powder (HEO-P), the same amounts of PVP (*M*_w_ ≈ 1.3 × 10^6^) and DMF were used, but calcined at 450 °C for 2 h.

*Fabrication of the low-entropy and medium-entropy oxide nanofibers:* Low-entropy and medium-entropy oxide nanofibers were prepared via electrospinning. The composition for Fe_2_O_3_ nanofibers included 0.7 g of PAN, 5.5 mL of DMF, and 1.5 mmol of Fe(NO_3_)_3_·9H₂O. The composition for Mn_3_O_4_ nanofibers included 0.7 g of PAN, 5.5 mL of DMF, and 2.5 mmol of (CH_3_COO)_2_Mn·4H₂O. The composition for Mn_3_O_4_ nanofibers included 0.7 g of PAN, 5.5 mL of DMF, and 2.5 mmol of (CH_3_COO)_2_Zn·2H₂O. The composition for Cr_2_O_3_/ZnO nanofibers included 0.7 g of PAN, 5.5 mL of DMF, 0.125 mmol of (CH_3_COO)_3_Cr and (CH_3_COO)_2_Zn·2H₂O. The composition for (MnCoZn)_3_O_4_ nanofibers included 0.7 g of PAN, 5.5 mL of DMF, 0.833 mmol of (CH_3_COO)_2_Mn·4H₂O, (CH_3_COO)_2_Co·4H₂O, and (CH_3_COO)_2_Zn·2H₂O. The composition for (MnFeCo)_3_O_4_ nanofibers included 0.7 g of PAN, 5.5 mL of DMF, 0.833 mmol of (CH_3_COO)_2_Mn·4H₂O, (CH_3_COO)_2_Co·4H₂O, and Fe(NO_3_)_3_·9H₂O. The composition for (MnFeCoZn)_3_O_4_ nanofibers included 0.7 g of PAN, 5.5 mL of DMF, 0.625 mmol of (CH_3_COO)_2_Mn·4H₂O, (CH_3_COO)_2_Co·4H₂O, Fe(NO_3_)_3_·9H₂O, and Fe(NO_3_)_3_·9H₂O. The composition for (CrMnCoZn)_3_O_4_ nanofibers included 0.7 g of PAN, 5.5 mL of DMF, 0.625 mmol of (CH_3_COO)_2_Mn·4H₂O, (CH_3_COO)_2_Co·4H₂O, (CH_3_COO)_3_Cr, and Fe(NO_3_)_3_·9H₂O. The electrospinning was conducted at a flow rate of 0.3 mL/h and a voltage of 12.5 kV. After calcination in air at 700 °C for 30 min, the composite nanofibers transformed into the low-entropy and medium-entropy oxide nanofibers.

*Fabrication of the Al_2_O_3_ nanofibers:* The Al(acac)_3_/PVP composite nanofibers were prepared by electrospinning a precursor containing 0.3 g of Al(acac)_3_, 0.3 g of PVP (*M*_w_ ≈ 1.3 × 10^6^), 2 mL of ethanol and 3 mL of acetone with a flow rate of 0.3 mL/h, at 15 kV. The as-spun Al(acac)_3_/PVP nanofibers were kept in air overnight and then converted to Al_2_O_3_ nanofibers after a calcination at 600 °C for 2 h in air with a ramping rate of 2.7 °C/min.

*Deposition of Pt nanoparticles on porous high-entropy oxide nanofibers:* Pt nanoparticles with a size below 3 nm were prepared by the classical polyol reduction method.^[S1]^ A simple impregnation method was used to load Pt nanoparticles onto the surface of nanofibers. Firstly, 25 mg of nanofibers were immersed in a mixture of 9 mL of ethanol and 1 mL of Pt suspension (0.575 mg/mL), then gently stirred for 2 hours. At a centrifugation speed of 7000 rpm/min, the catalysts were washed with ethanol five times for three minutes. Then, the Pt/HEO-*n* °C were heated in the N_2_ atmosphere at different temperatures for 2 h (*n* was the aging temperature).

*In Situ HAADF−STEM observation of thermal stability of Pt nanoparticles:* The selection of the electrical chip (E-chip)-based heating holder (NanoEx-i/v MEMS) was based on its minimal drift when heating. Before commencing the experiment, the settings have been calibrated to account for the slight variations in the thermal conductivity of each chip, hence preventing any temperature variance. During the *in situ* experiment, the sample underwent heating at a constant rate of 5 °C per second, followed by a 5-minute hold at each temperature interval. It is important to mention that the beam intensity used is minimal, ensuring safety and preventing any alteration in the morphology of the sample.

*Evaluation of the catalytic effect on CO oxidation:* The CO oxidation reaction was conducted in a flow-through fixed-bed quartz reactor system. The catalyst was pretreated under 10% H_2_/Ar (40 mL/min) at 200 °C for 1 h, before cooling down to room temperature. The steady-state CO oxidation activity was measured, with the duration time for each temperature point maintained as 10 min. The feeding stream consists of 1% CO and 5% O_2_ balanced with Ar. The total flow rate was controlled at 30 mL/min thus achieving the weight hourly space velocity (WHSV) of 90 000 mL∙g⁻^1^∙h⁻^1^. The outlet concentrations of CO and CO_2_ were analyzed using an online gas chromatograph (Shimadzu, Japan, 2014, SSZC1C01283) containing a thermal conductivity detector (TCD) and a flame ionization detector (FID) equipped with a methanizer.

*CO pulse chemisorption experiments:* Pt dispersion measurements were conducted on a VDsorb 91i, and the signal was collected using a thermal conductivity detector (TCD). The Pt/HEO sample was first treated in a flow of He at 200 °C for 1 h before measurement, followed by cooling to 50 °C. The sample was then purged for 40 minutes to establish a stable baseline. A 10% CO/He gas mixture was injected in pulses into the reactor until CO adsorption saturation was achieved. The volume of each pulse was 0.45 cm^3^.

*Characterizations:* Transmission electron microscopy (TEM) images were captured using a Tecnai G2 T20 microscope at 200 kV. Scanning electron microscopy (SEM) images were obtained with a FEI Nova Nano SEM 230. High-angle annular darkfield scanning transmission electron microscopy (HAADF−STEM) images were collected using a Talos F200X microscope. Crystal structure information was acquired through X-ray diffraction (XRD) with a Bruker D8 Advance using Cu-Kα radiation (λ = 1.5406 Å). XRD analysis was conducted with an Ultima IV at a continuous scan rate of 2°·min⁻¹ in the 5–90° range at room temperature. The Brunauer–Emmett–Teller (BET) specific surface area was measured using an Autosorb-iQ (Quantachrome). X-ray photoelectron spectroscopy (XPS) measurements were performed with a Thermo-VG Scientific Esea Lab 250. CO pulse chemisorption experiments were conducted on VDsorb 91i and the signal was collected with a thermal conductivity detector (TCD).


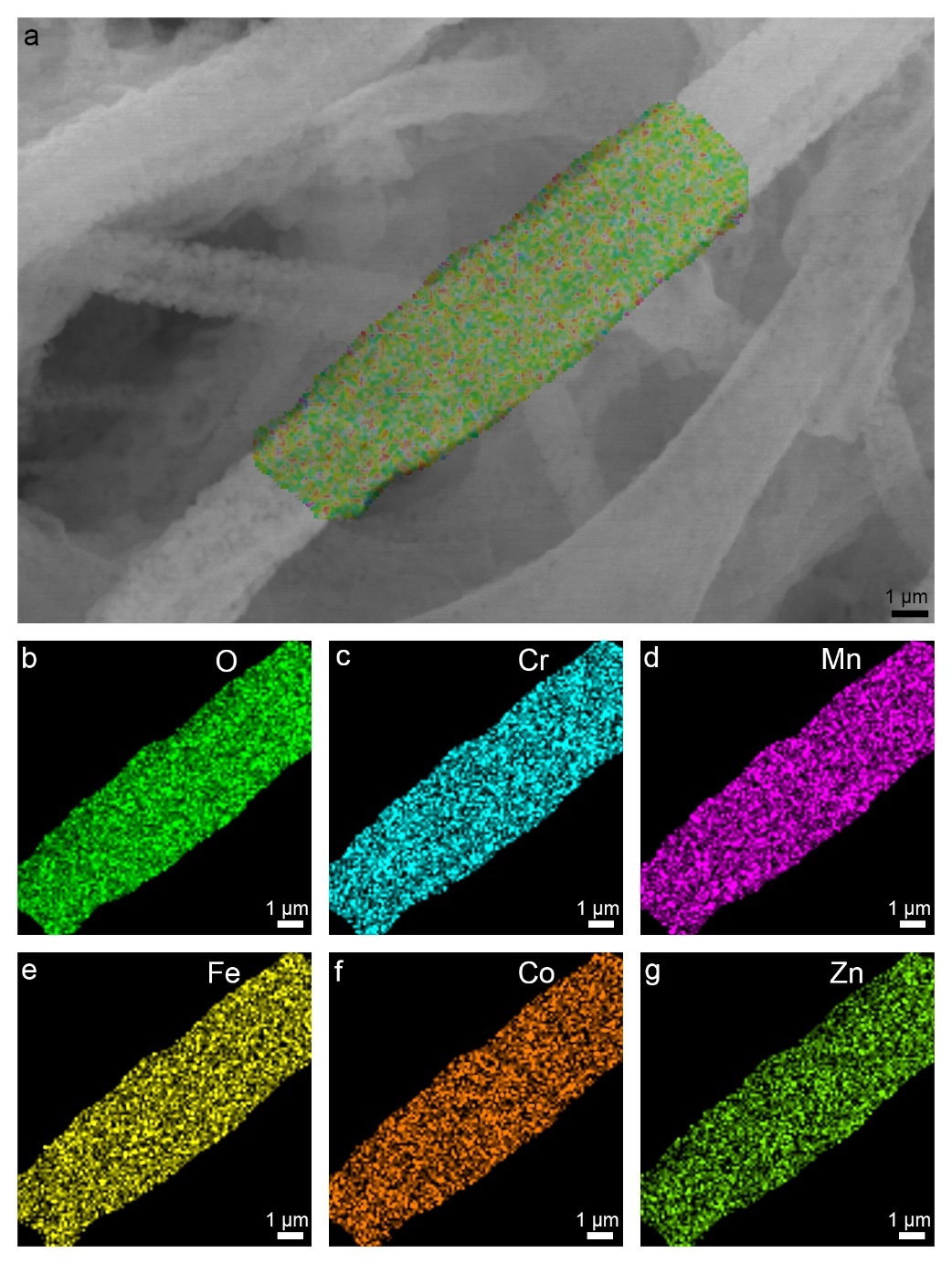


**Figure S1.** (a) SEM image and (b-g) EDS elements mapping images of the high-entropy oxide nanofibers.


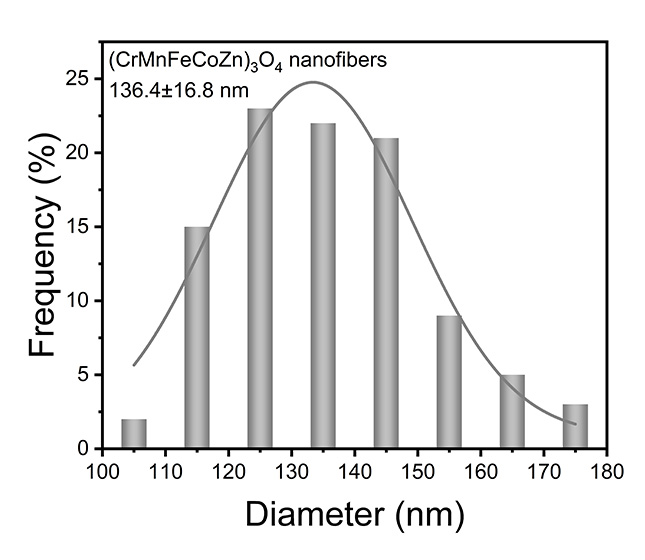


**Figure S2.** The diameter distribution of the high-entropy oxide nanofibers.


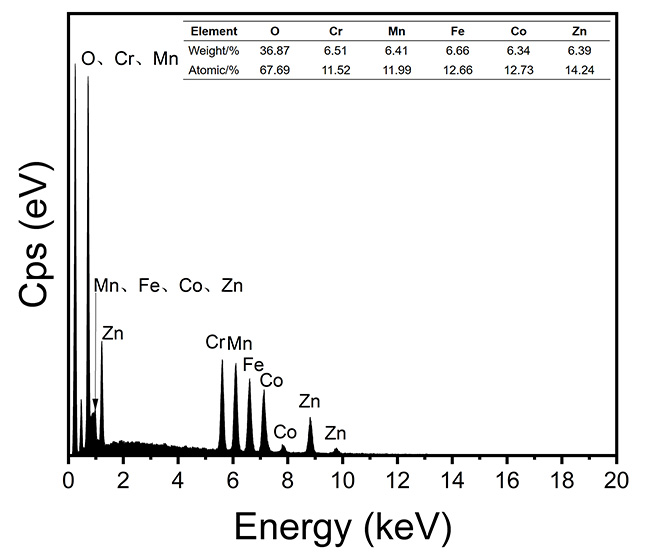


**Figure S3.** EDS data of the HEO nanofibers.


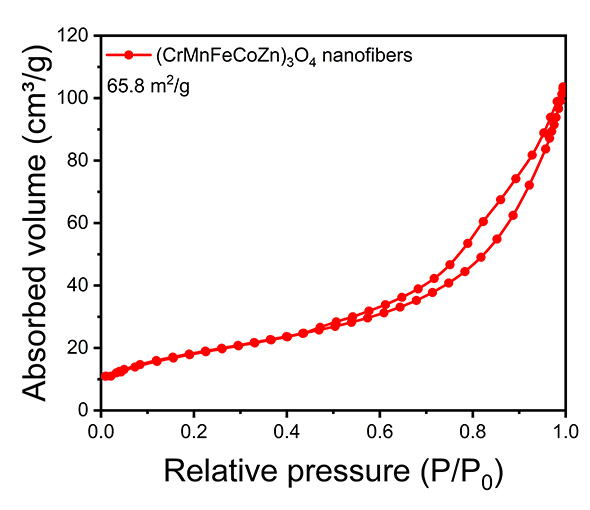


**Figure S4.** N_2_ adsorption/desorption isotherms of the HEO nanofibers.


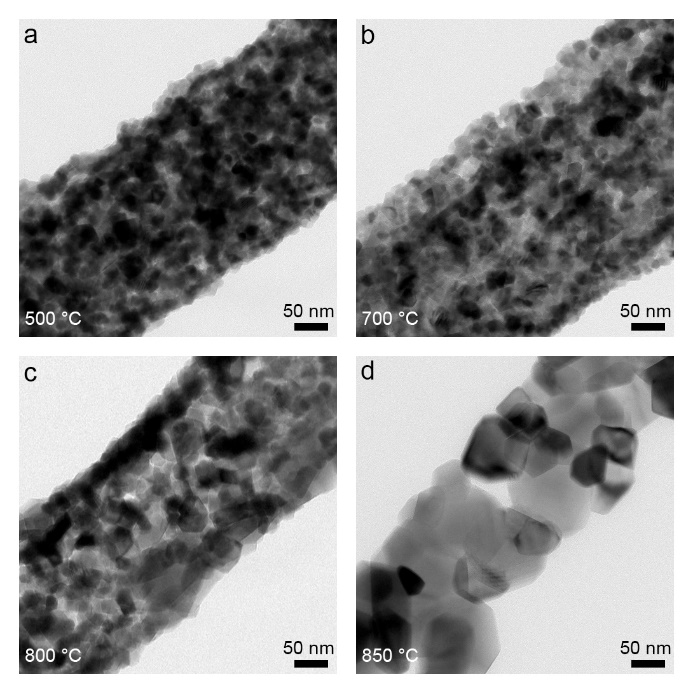


**Figure S5.** TEM images of the HEO nanofibers after being aged at elevated temperatures.


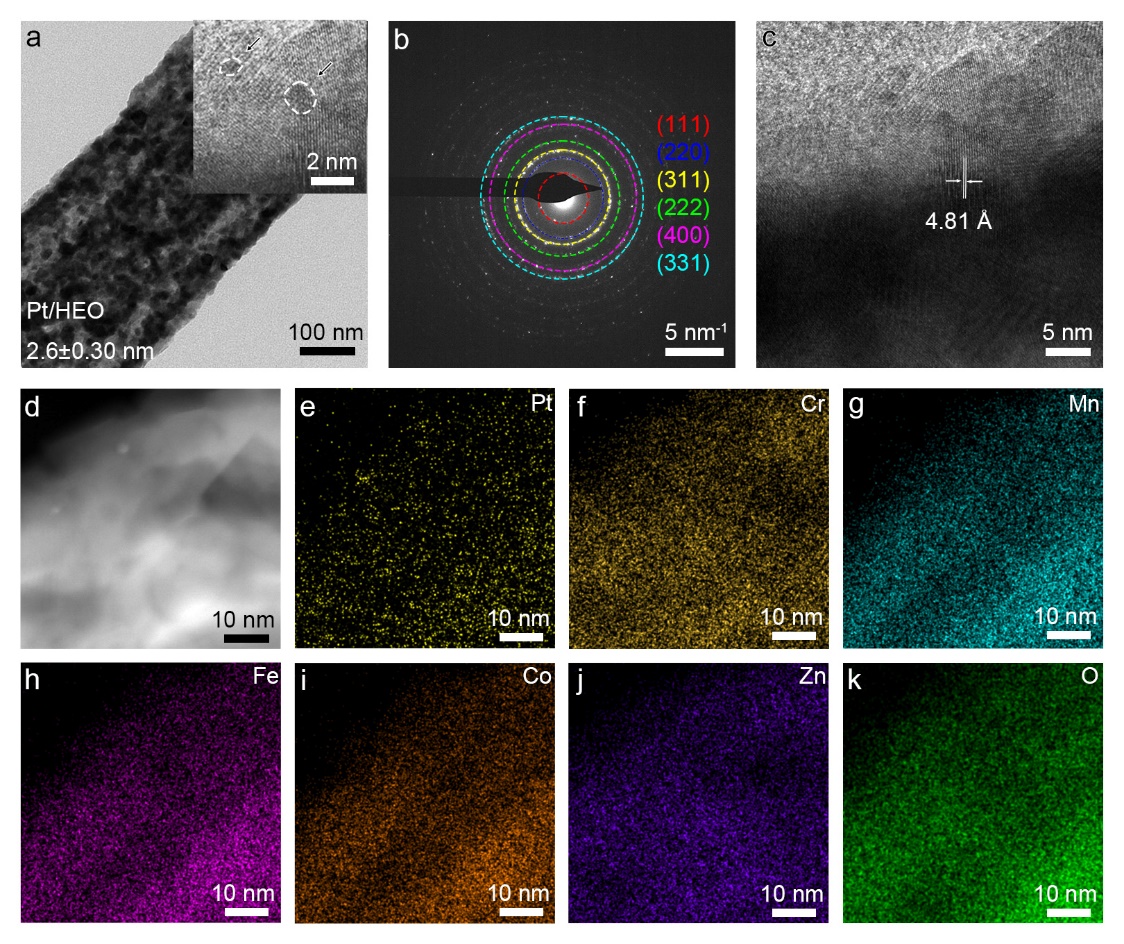


**Figure S6.** (a) TEM image of Pt/HEO. (b) The corresponding SEAD pattern of Pt/HEO. (c) HRTEM image of Pt/HEO. (d) HAADF−STEM image and (e-k) EDS elements mapping images of Pt/HEO.


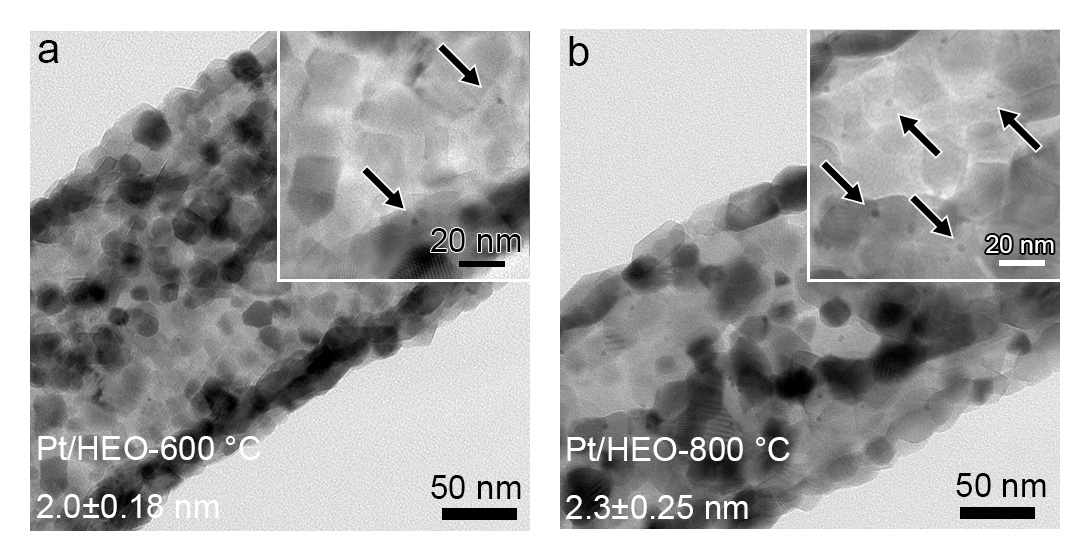


**Figure S7.** TEM images of (a) Pt/HEO-600 °C and (b) Pt/HEO-800 °C.


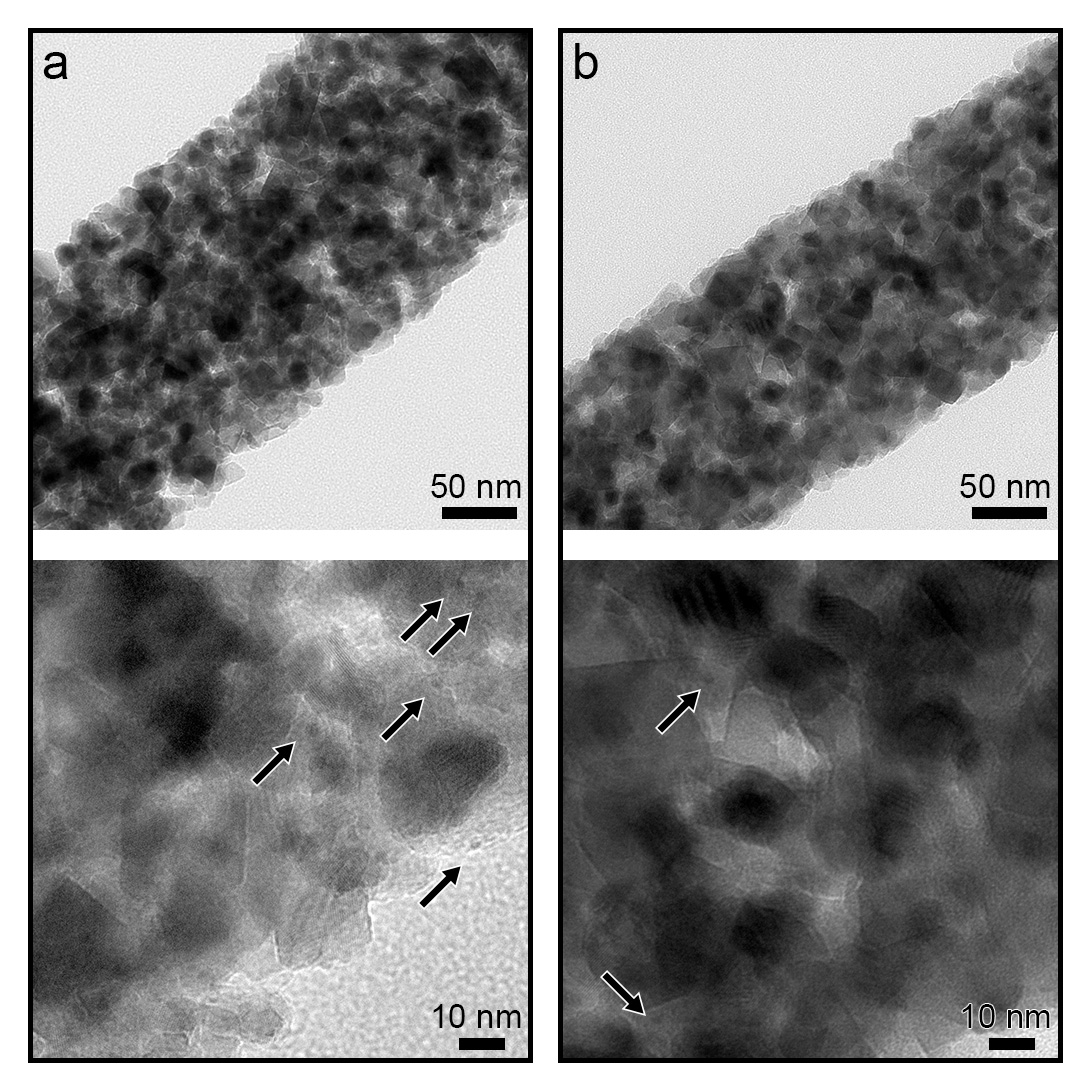


**Figure S8.** TEM images of (a) Pt/HEO-500 °C, (b) Pt/HEO-600 °C in air.


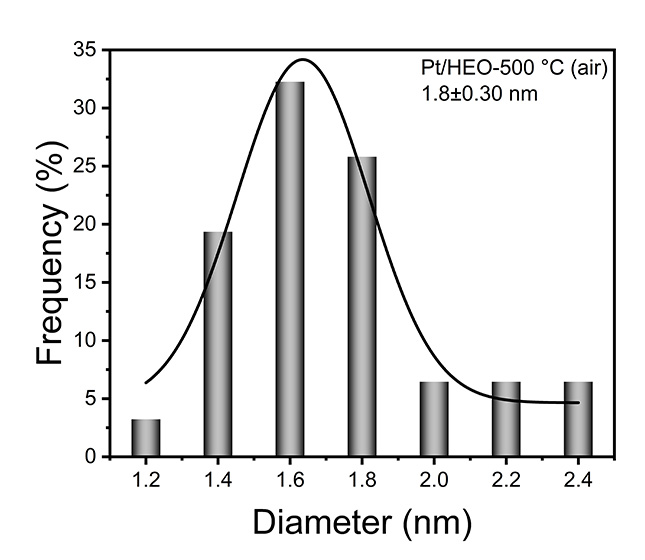


**Figure S9.** The size distribution histogram of Pt nanoparticles on HEO after aging at 500 °C in air.


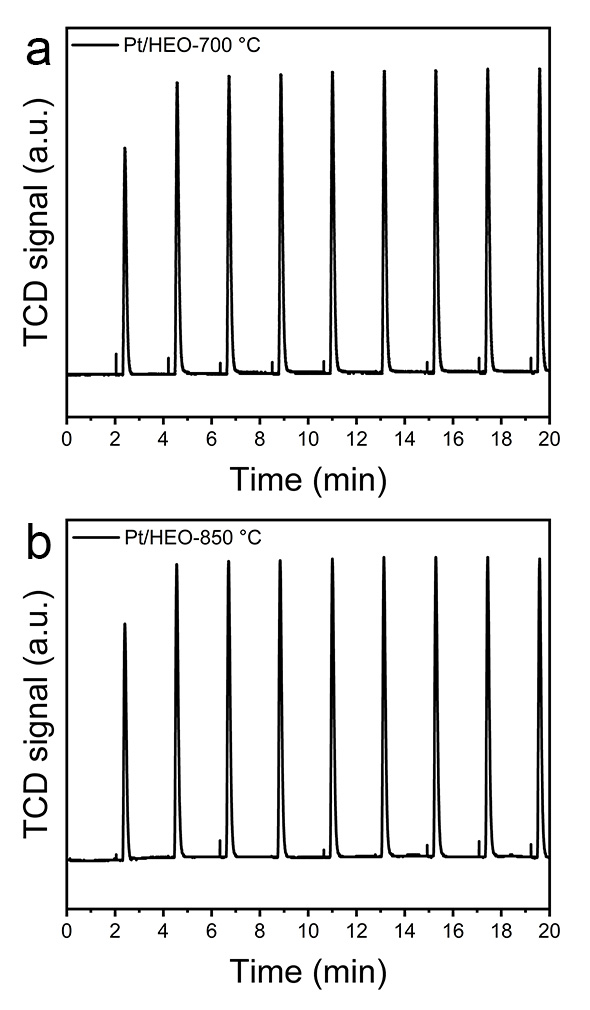


**Figure S10.** CO pulse chemisorption results of (a) Pt/HEO-700 °C and (b) Pt/HEO-850 °C.


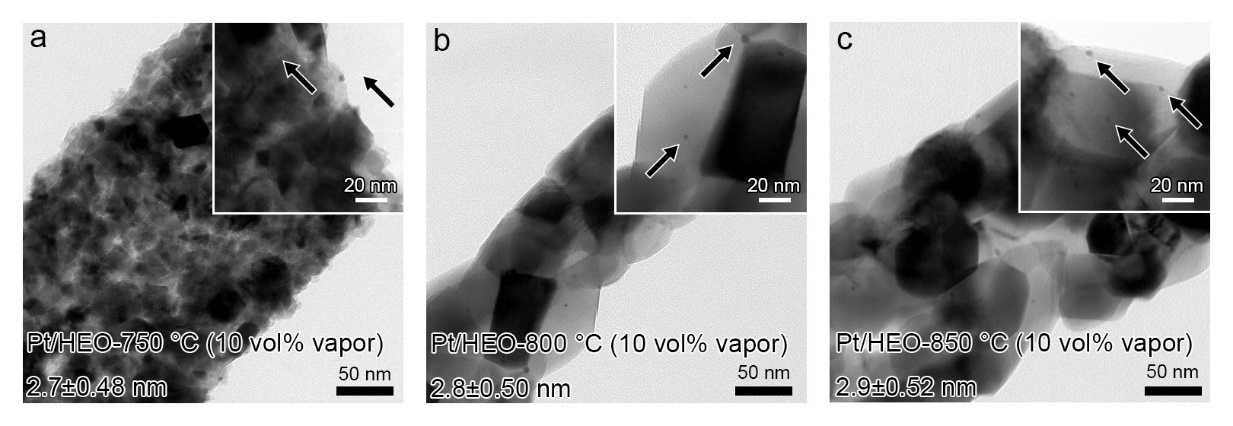


**Figure S11.** TEM images of (a) Pt/HEO-750 °C (10 vol% vapor), (b) Pt/HEO-800 °C (10 vol% vapor) and (c) Pt/HEO-850 °C (10 vol% vapor).


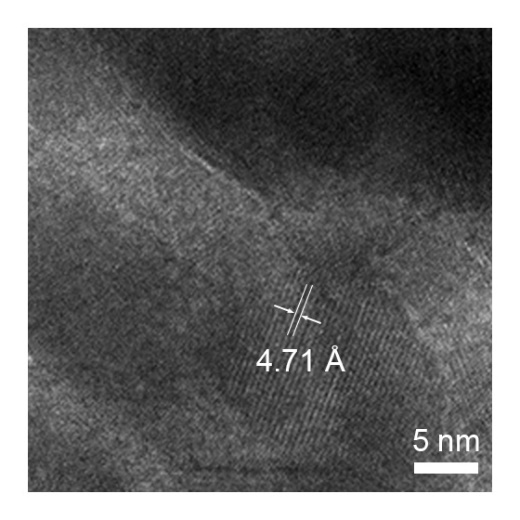


**Figure S12.** HRTEM image of Pt/HEO-700 ℃.


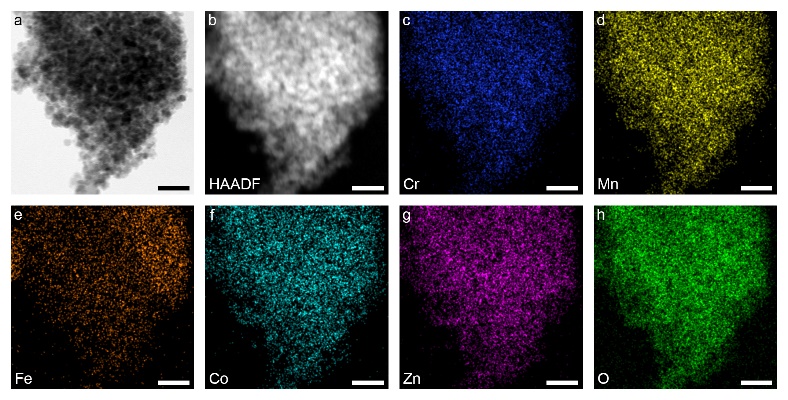


**Figure S13.** HAADF−STEM image and elemental mappings of the (CrMnFeCoZn)_3_O_4_ HEO-P. The scale bars are 50 nm.


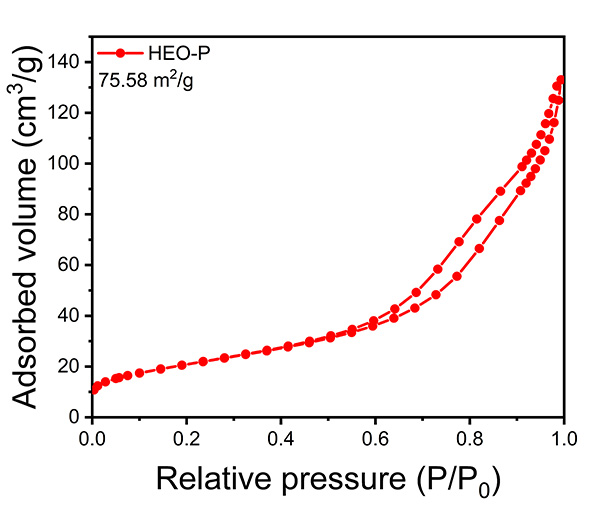


**Figure S14.** N_2_ adsorption/desorption isotherms of the HEO-P.

**Table S1.** ζ-potential of Pt nanoparticles, HEO nanofibers and HEO-P

| Sample | ζ-potential/ mV | | | |
| --- | --- | --- | --- | --- |
|  | 1 | 2 | 3 | average |
| Pt nanoparticles | 1.2 | 2.0 | 0.9 | 1.40 |
| HEO nanofibers | －0.75 | 0.83 | －0.042 | 0.013 |
| HEO-P | －0.60 | －0.67 | －0.46 | －0.58 |


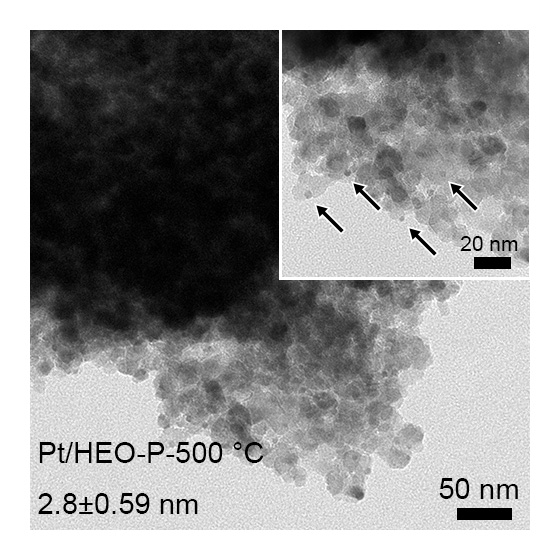


**Figure S15.** TEM image of Pt/HEO-P-500 °C.


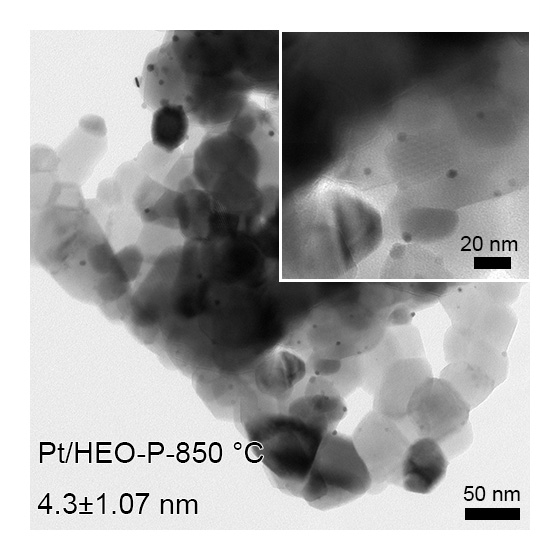


**Figure S16.** TEM image of Pt/HEO-P-850 °C.


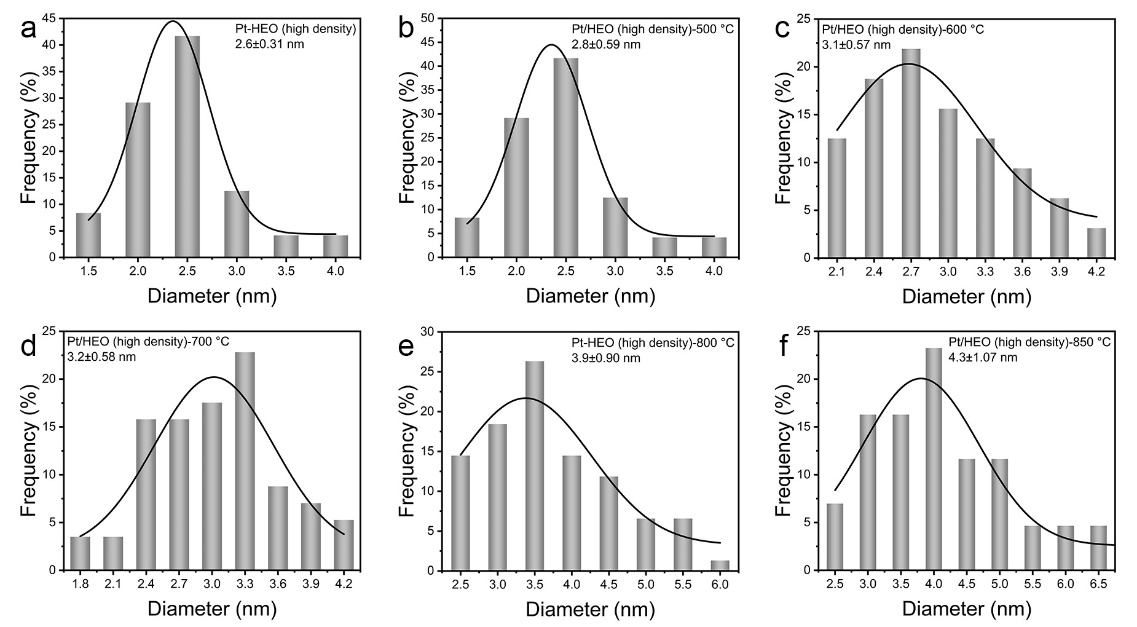


**Figure S17.** The size distribution histogram of Pt nanoparticles on HEO-P at elevated temperatures.


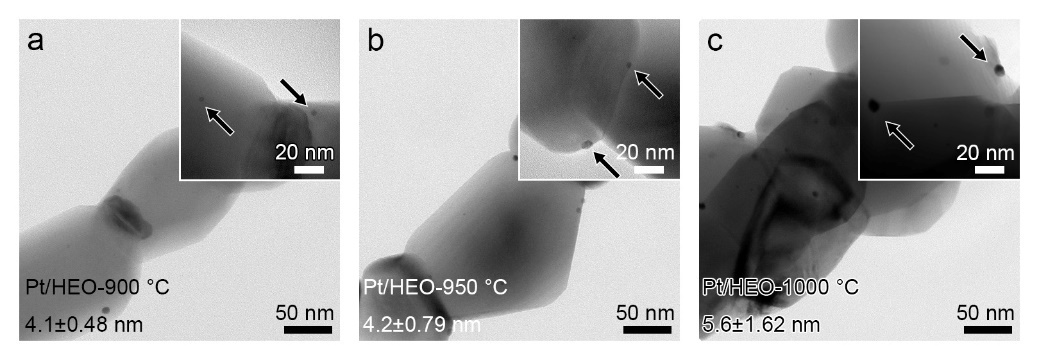


**Figure S18.** TEM images of (a) Pt/HEO-900 °C, (b) Pt/HEO-950 °C and (c) Pt/HEO-1000 °C.


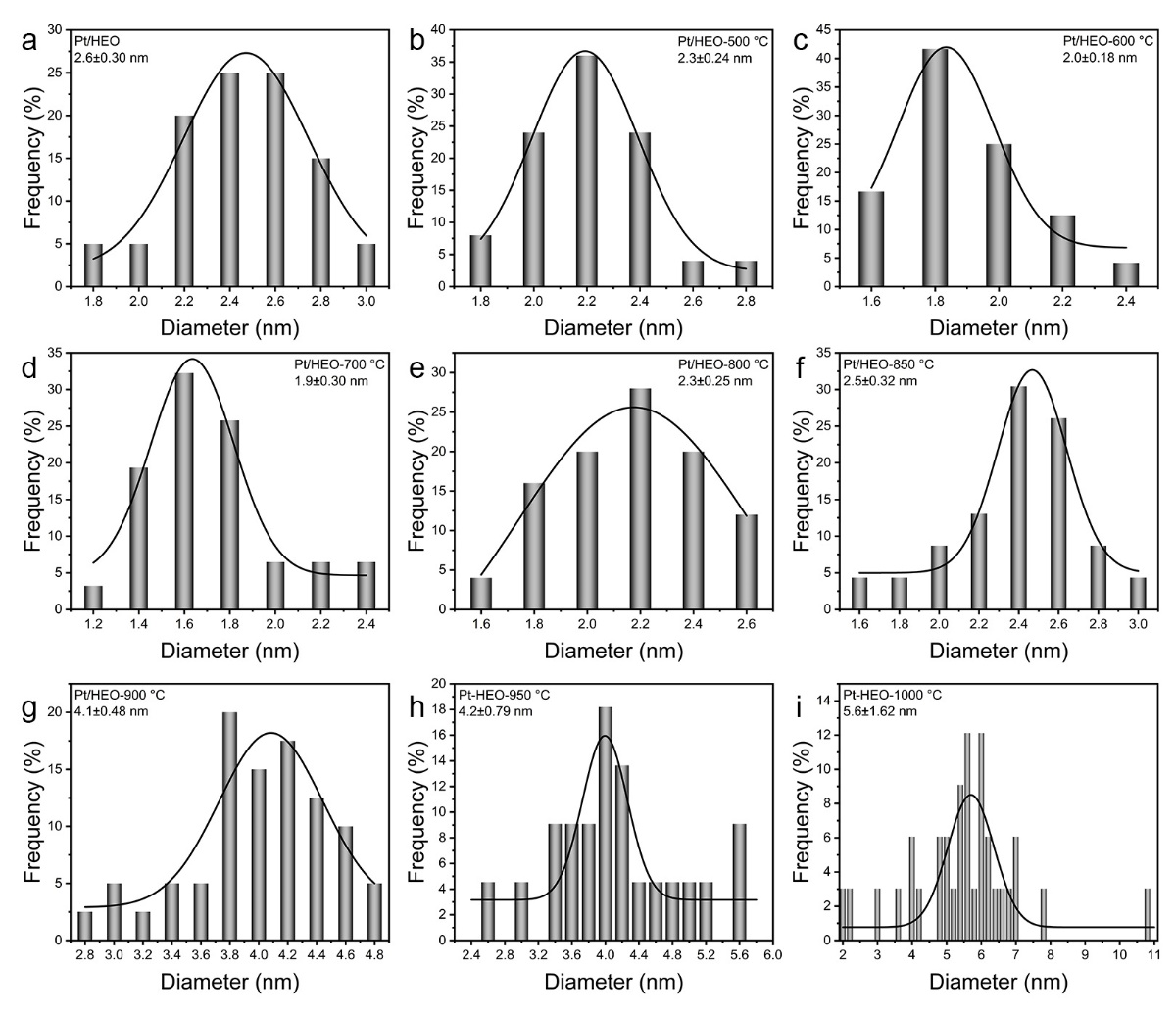


**Figure S19.** The size distribution histograms of Pt nanoparticles on HEO nanofibers at elevated temperatures in N_2_.


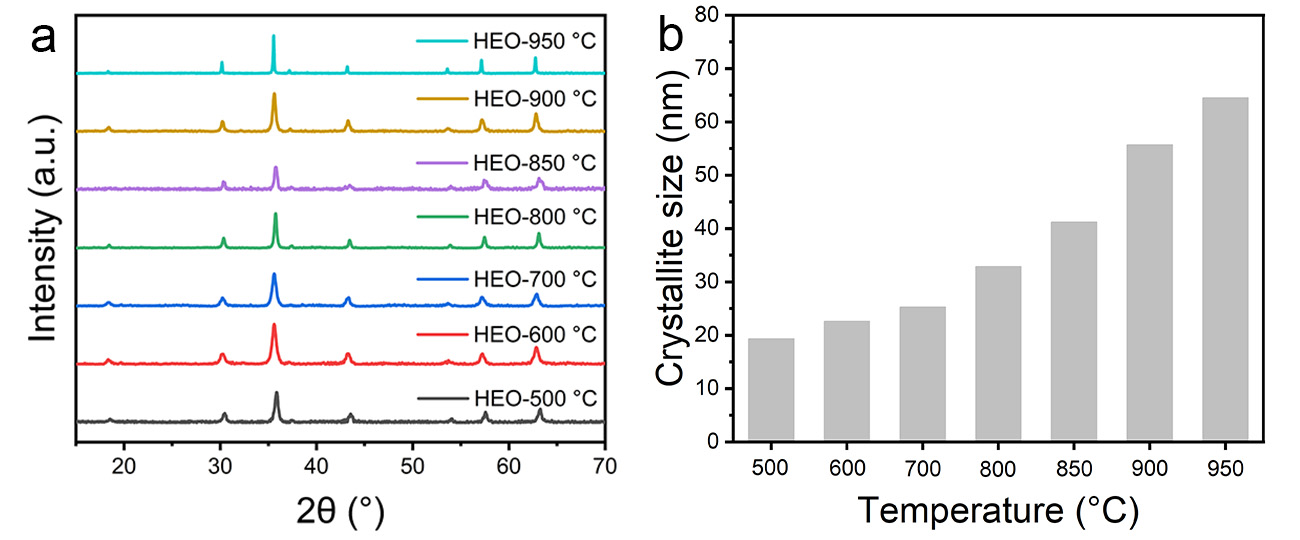


**Figure S20.** (a) XRD patterns of (CrMnFeCoZn)_3_O_4_ nanofibers at elevated temperatures. (b) The calculated average nanocrystal size on the base of Scherrer equation.


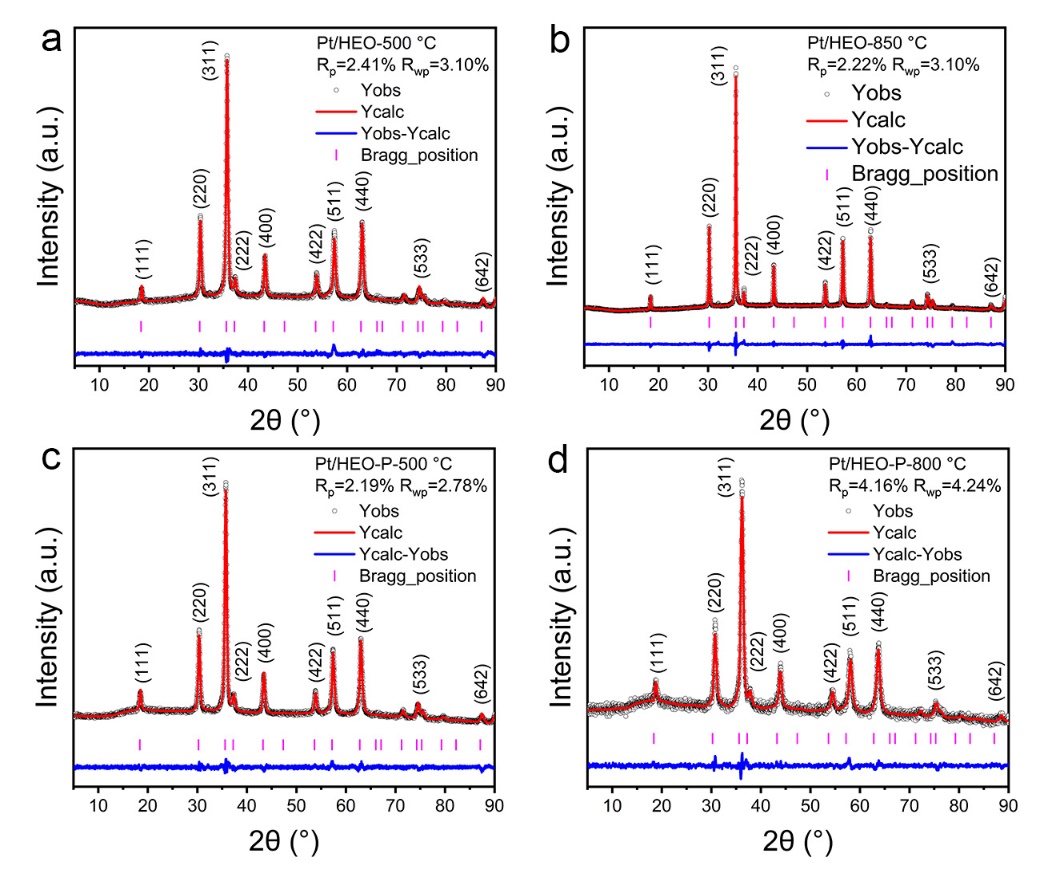


**Figure S21.** Rietveld refinement patterns of (a) Pt/HEO-500 °C, (b) Pt/HEO-850 °C, (c) Pt/HEO-P-500 °C, and (d) Pt/HEO-P-800 °C display the observed data points (red circles), the calculated intensity (black line), the positions of reflection peaks (pink vertical bars), and the difference profile (blue line).


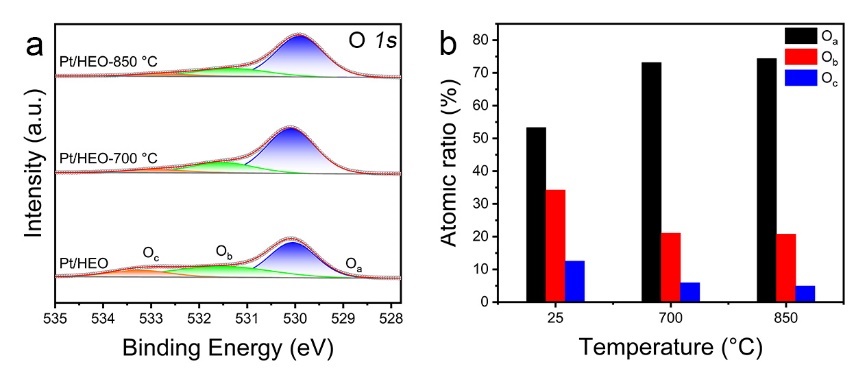


**Figure S22.** (a, b) O *1s* XPS spectra of Pt/HEO catalytic system after being aged at elevated temperatures.


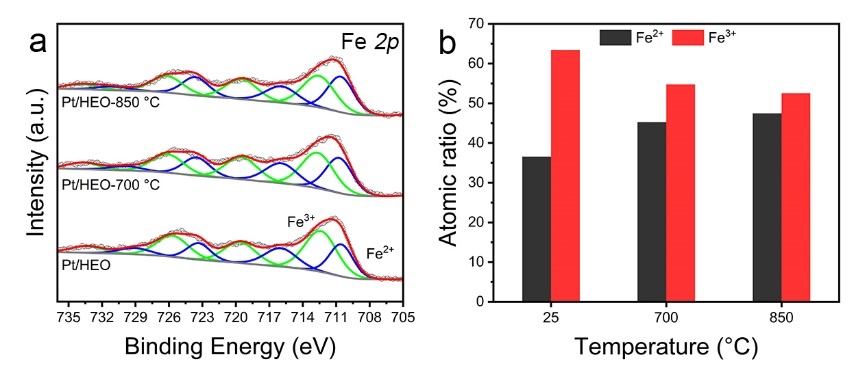


**Figure S23.** (a, b) Fe *2p* XPS spectra of Pt/HEO nanofibers after being aged at elevated temperatures.


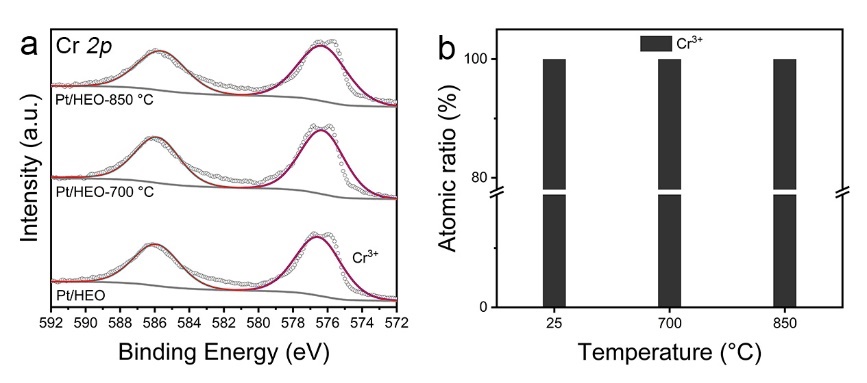


**Figure S24.** (a, b) Cr *2p* XPS spectra of Pt/HEO after being aged at elevated temperatures.


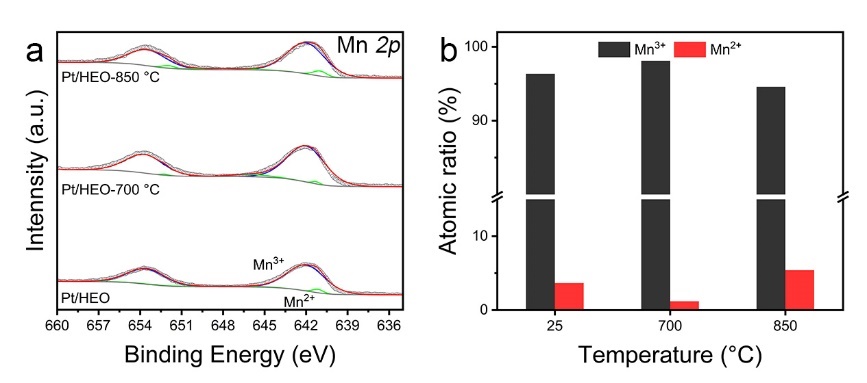


**Figure S25.** (a, b) Mn *2p* XPS spectra of Pt/HEO after being aged at elevated temperatures.


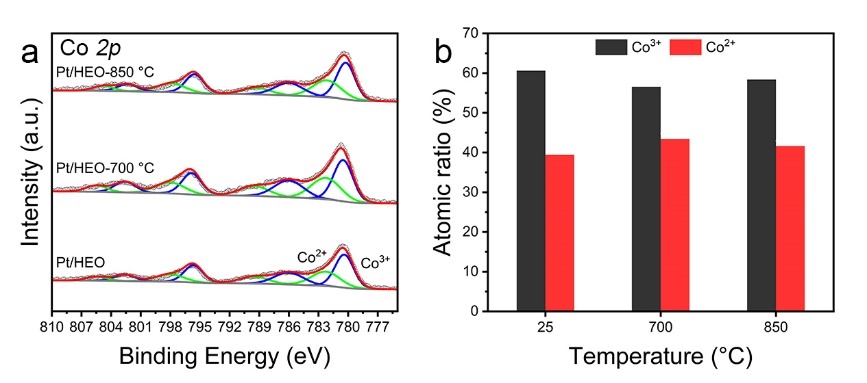


**Figure S26.** (a, b) Co *2p* XPS spectra of Pt/HEO after being aged at elevated temperatures.


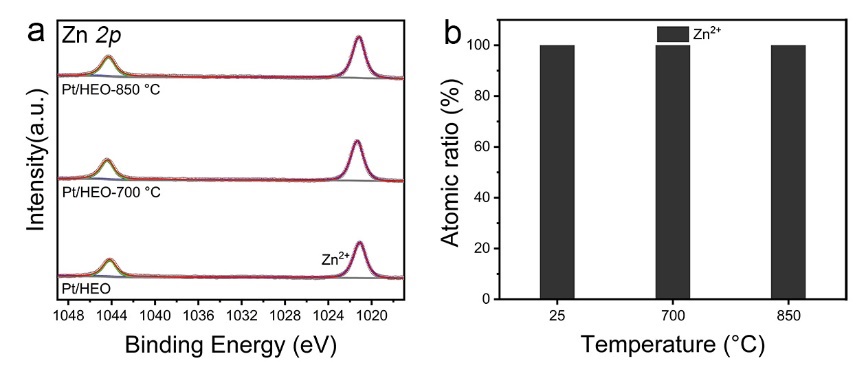


**Figure S27.** (a, b) Zn *2p* XPS spectra of Pt/HEO after being aged at elevated temperatures.


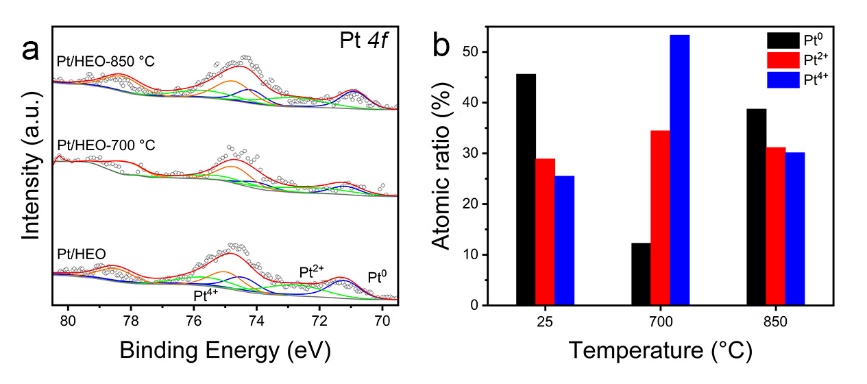


**Figure S28.** (a, b) Pt *4f* XPS spectra of Pt/HEO after being aged at elevated temperatures.


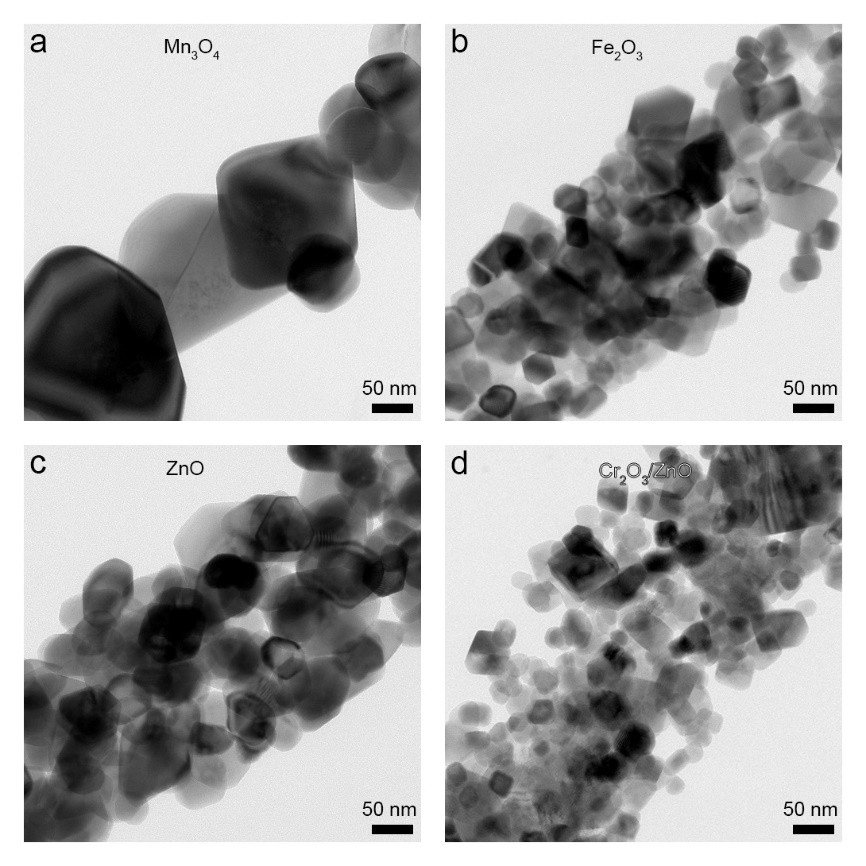


**Figure S29.** TEM images of the low-entropy oxide nanofibers.


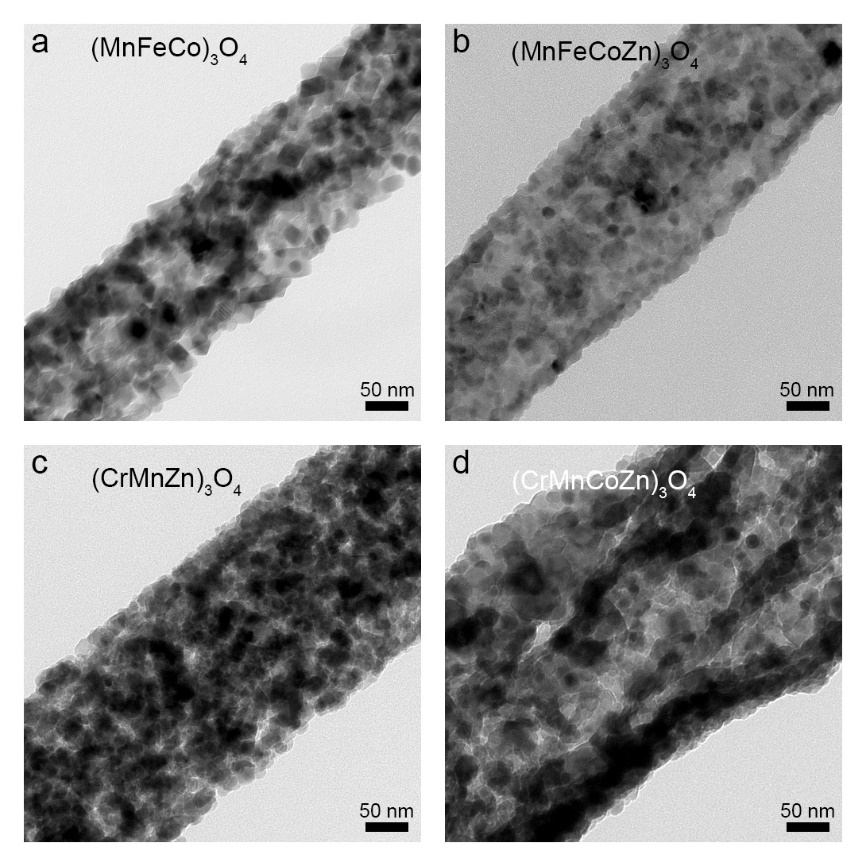


**Figure S30.** TEM images of medium-entropy oxide nanofibers.


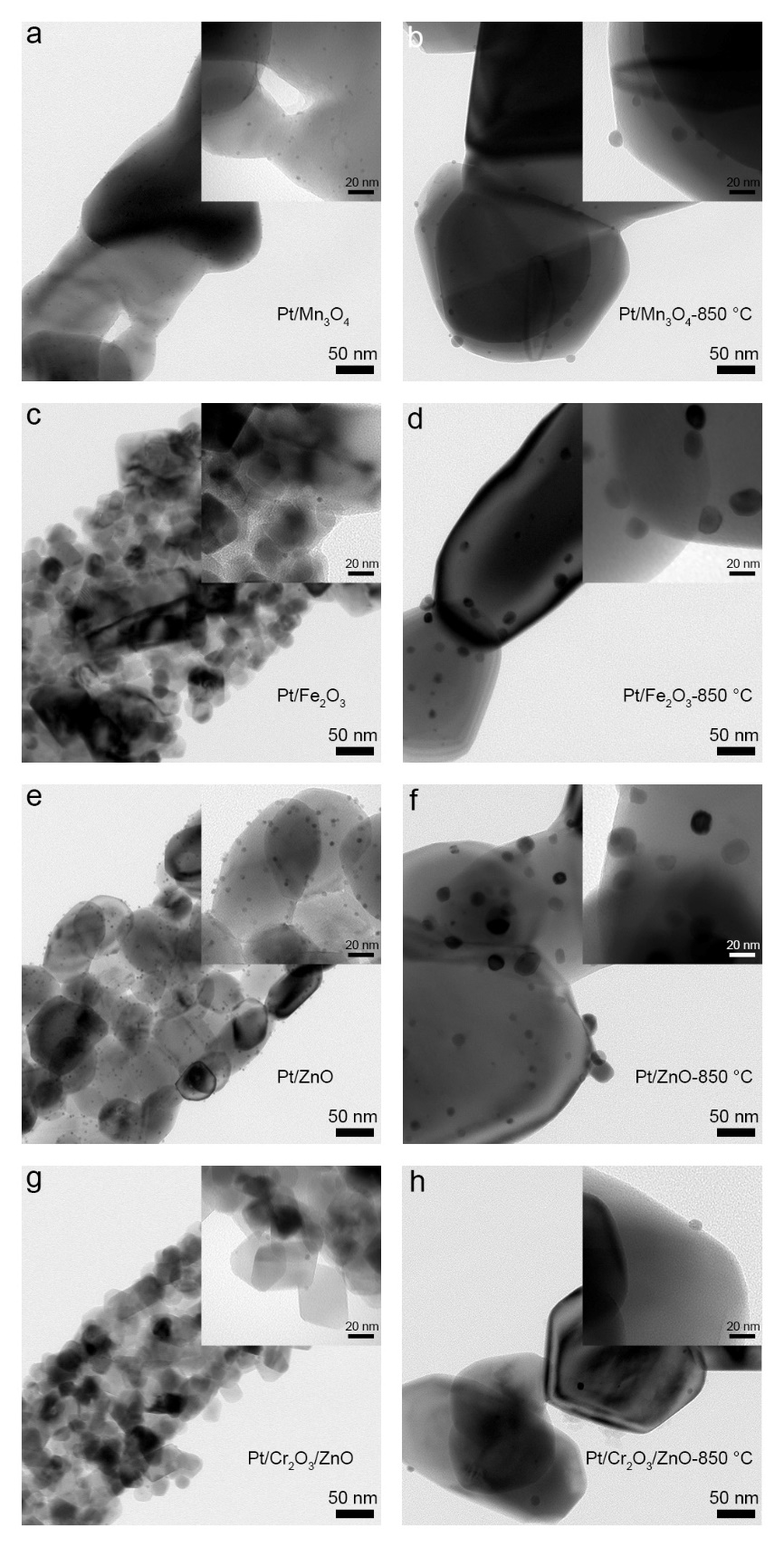


**Figure S31.** TEM image of Pt/low-entropy oxide nanofibers after being aged at elevated temperatures.


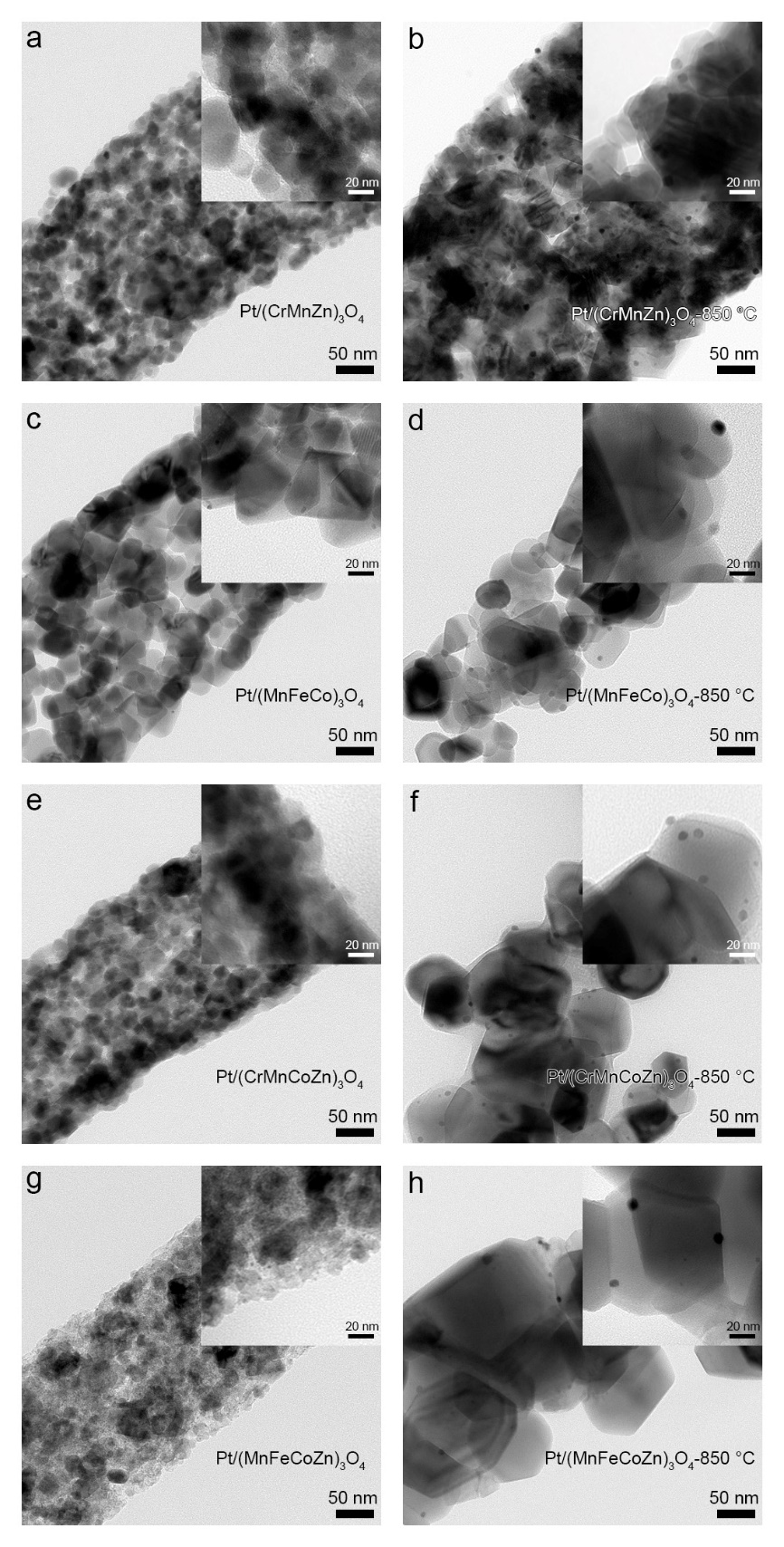


**Figure S32.** TEM image of Pt/medium-entropy oxide nanofibers after being aged at elevated temperatures.

**Table S2.** Comparison of size of metal catalysts

| **Materials** | **Temperature/°C** | **Size of metal catalysts** | **Reference** |  |
| --- | --- | --- | --- | --- |
|  |  |  |  |  |
| Pt/CeO_2_ | 600 | 7.47 | [S2] |  |
| Pt/Al_2_O_3_/TiO_2_ | 600 | 4.73 | [S3] |  |
| Co_3_O_4_/Pt/Al_2_O_3_ | 600 | 2.91 | [S4] |  |
| Pt@CeO_2_ | 700 | 5.50 | [S5] |  |
| Pt/γ-Al_2_O_3_-h-BN | 700 | 9.20 | [S6] |  |
| AuPd/CeO_2_ZrO_2_ | 700 | 6.40 | [S7] |  |
| Au/P_25_ | 700 | 8.40 | [S8] |  |
| Au@SiO_2_/TiO_2_ | 800 | 6.40 | [S9] |  |
| Pt-Ga_2_O_3_/S_40_ | 800 | 35.6 | [S10] |  |
| Pt/HEO | 800 | 2.26 | This work |  |
| Pt/HEO | 850 | 2.53 | This work |  |
| Pt/HEO | 950 | 4.23 | This work |  |


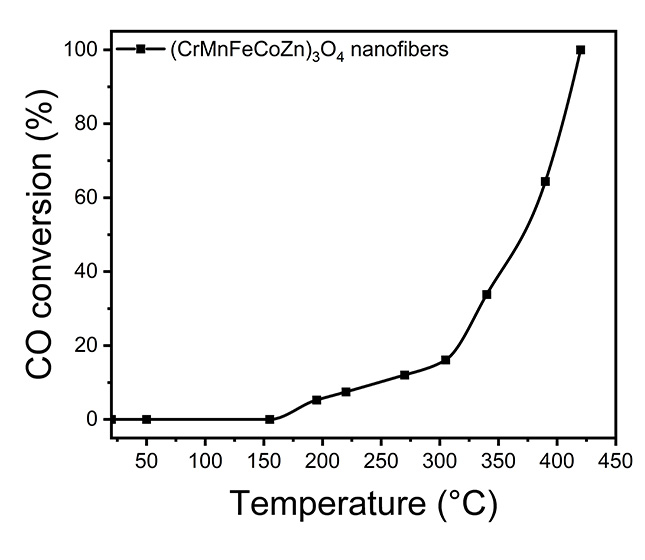


**Figure S33.** CO oxidation performance of the HEO nanofibers.


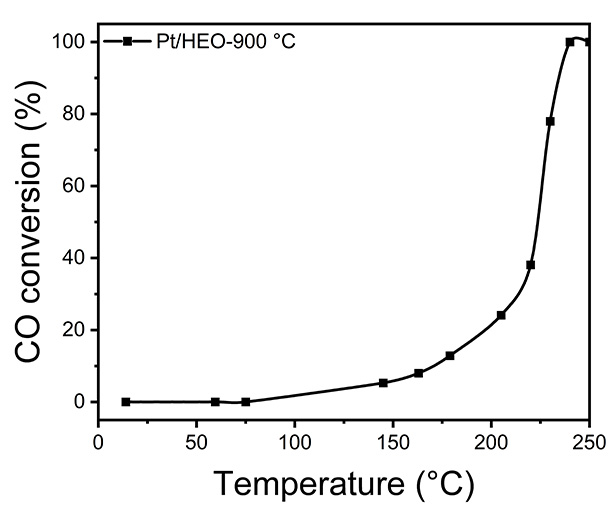


**Figure S34.** CO oxidation performance of Pt/HEO at 900 °C.


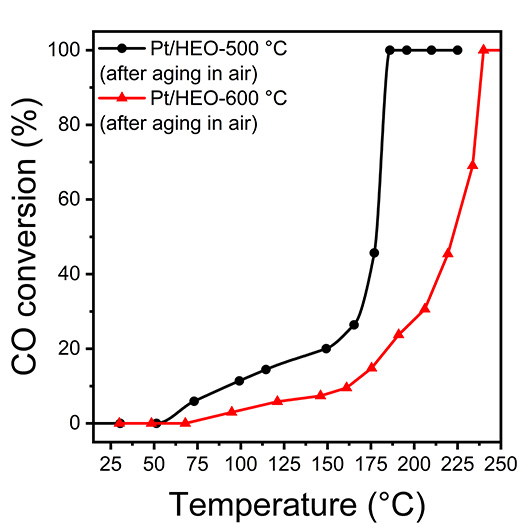


**Figure S35.** CO oxidation performance of Pt/HEO in air after being aged at elevated temperatures.

**Table S3.** Comparison of the long-term cycling performance of different catalytic systems for CO oxidation

| **Materials** | **Time/h** | **Reference** |  |
| --- | --- | --- | --- |
|  |  |  |  |
| Pt/Sn_0.2_Ti_0.8_O_2_ | 168 | [S11] |  |
| Pd/CeO_2_ | 100 | [S12] |  |
| Pt/CeO_2_ | 300 | [S13] |  |
| 5Cu−50CeO_2_@SiO_2_ | 36 | [S14] |  |
| Ce_0.8_Cu_0.2_O_2−δ_ | 72 | [S15] |  |
| Pd/TiO_2_-A400 | 240 | [S16] |  |
| Pd_1_@(CeZrHfTiLa)O_x_ | 100 | [S17] |  |
| Pt/HEO-850 °C | 338 | This work |  |


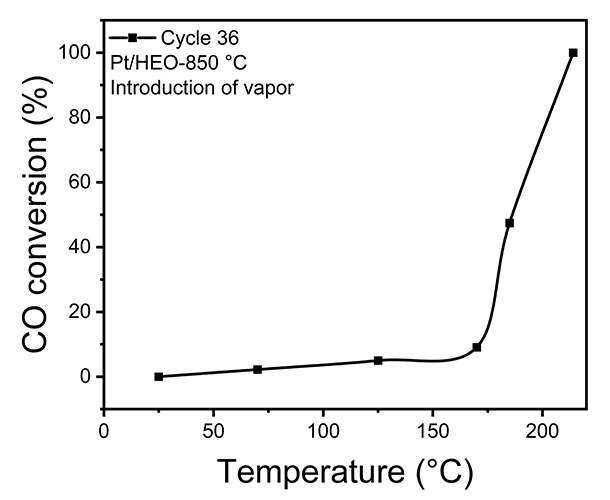


**Figure S36.** The CO oxidation performance was evaluated during the 36^th^ cycle with the introduction of vapor.


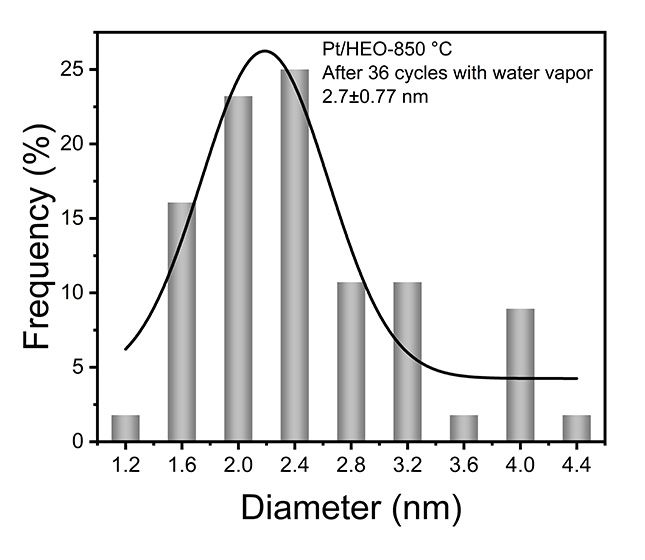


**Figure S37.** The size distribution histogram of Pt nanoparticles on Pt/HEO-850 °C after 36 cycles with vapor.


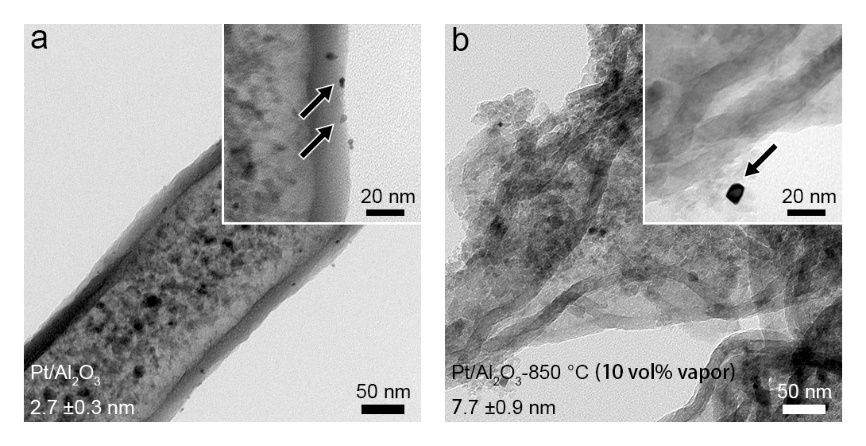


**Figure S38.** TEM images of (a) Pt/Al_2_O_3_ and (b) Pt/Al_2_O_3_-850 °C (10 vol% vapor).


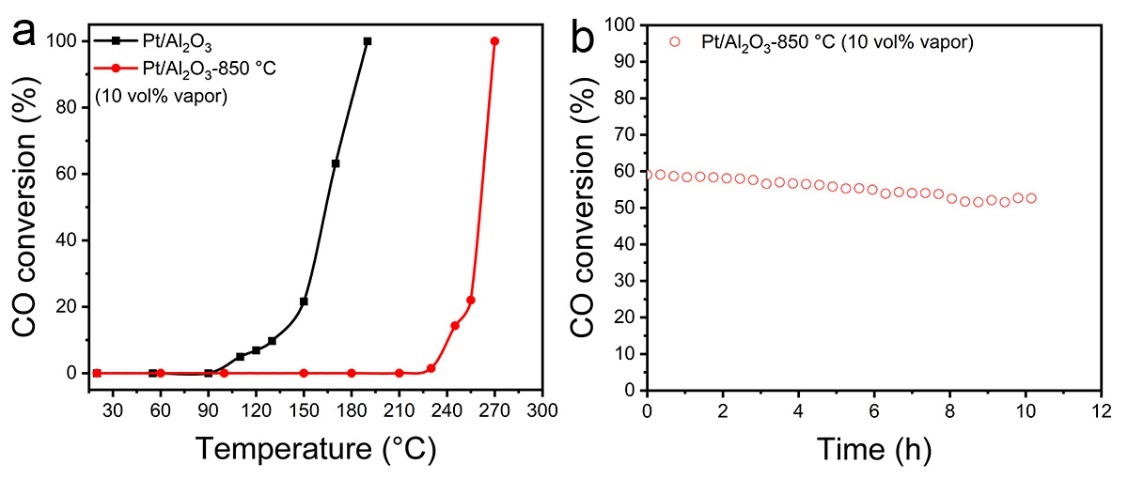


**Figure S39.** (a) CO oxidation performance of Pt/Al_2_O_3_ and Pt/Al_2_O_3_-850 °C (10 vol% vapor). (b) The stability of Pt/Al_2_O_3_-850 °C (10 vol% vapor) assessed during prolonged operation with sustained 59% CO conversion.

References

[S1] W. Fu, K. Yin, Z. Li, J. Wang, M. Tang, J. Tian, L. Sun, Y. Sun, Y. Dai, *Chem. Sci.* **2024**, *15*, 16688.

[S2] J. Wang, S. Liu, M. Tang, W. Fu, Y. Wang, K. Yin, Y. Dai, *Small* **2023**, *19*, e2300547.

[S3] W. Fu, Z. Li, W. Xu, Y. Wang, Y. Sun, Y. Dai, *Mater. Today Nano* **2020**, *11*, 100088.

[S4] X. Liu, Q. Zhu, Y. Lang, K. Cao, S. Chu, B. Shan, R. Chen, *Angew. Chem. Int. Ed.* **2017**, *56*, 1648.

[S5] M. Tang, S. Liu, W. Fu, J. Wang, K. Yin, M. Zhu, J. Tian, Y. Sun, Y. Dai, *Mater. Today Nano* **2022**, *20*, 100249.

[S6] S. Wang, Z. Li, M. Yang, Y. Li, R. Li, C. Yu, Y. Wang, Y. Jiang, T. Li, J.-X. Liu, H. Zhang, Z. Zhao, C. Xu, G. Jiang, *Sci. China Mater.* **2021**, *64*, 1930.

[S7] C. M. Olmos, L. E. Chinchilla, A. Villa, J. J. Delgado, A. B. Hungría, G. Blanco, L. Prati, J. J. Calvino, X. Chen, *J. Catal.* **2019**, *375*, 44.

[S8] Y. Tang, X. Ma, X. Du, X. Liu, R. Chen, B. Shan, *J. Catal.* **2023**, *423*, 145.

[S9] Y. Zhang, J. Zhang, B. Zhang, R. Si, B. Han, F. Hong, Y. Niu, L. Sun, L. Li, B. Qiao, K. Sun, J. Huang, M. Haruta, *Nat. Commun.* **2020**, *11*, 558.

[S10] G. Ren, S. Xiong, X. Li, X. Lai, J. Chen, M. Chu, Y. Xu, S. Huang, *J. Catal.* **2024**, *429*, 115276.

[S11] J. Chen, S. Xiong, H. Liu, J. Shi, J. Mi, H. Liu, Z. Gong, L. Oliviero, F. Mauge, J. Li, *Nat. Commun.* **2023**, *14*, 3477.

[S12] Y. Deng, P. Tian, S. Liu, H. He, Y. Wang, L. Ouyang, S. Yuan, *J. Hazard. Mater.* **2022**, *426*, 127793.

[S13] Y. Xiao, H. Li, K. Xie, *Angew. Chem. Int. Ed.* **2021**, *60*, 5240.

[S14] Y. Y. Song, L. Y. Du, W. W. Wang, C. J. Jia, *Langmuir* **2019**, *35*, 8658.

[S15] A. F. Zedan, K. Polychronopoulou, A. Asif, S. Y. AlQaradawi, A. S. AlJaber, *Surf. Coat. Tech.* **2018**, *354*, 313.

[S16] J. Chen, Y. Su, Q. Meng, H. Qian, L. Shi, J. A. Darr, Z. Wu, X. Weng, *Angew. Chem. Int. Ed.* **2023**, *62*, e202310191.

[S17] H. Xu, Z. Zhang, J. Liu, C. L. Do-Thanh, H. Chen, S. Xu, Q. Lin, Y. Jiao, J. Wang, Y. Wang, Y. Chen, S. Dai, *Nat. Commun.* **2020**, *11*, 3908.
